# Obesity-driven low-grade chronic inflammation as a mechanistic bridge to chronic pain: from adipose tissue remodeling to central sensitization

**DOI:** 10.3389/fimmu.2026.1889437

**Published:** 2026-07-30

**Authors:** Yi-xin Ma, Meng Lin, Yang Nan, Nan-nan Li, Bing Liu, Hao-ran Wang, Shun-yu Ning, Zhe Zhang

**Affiliations:** 1 Liaoning University of Traditional Chinese Medicine, Shenyang, Liaoning, China; 2 Affiliated Hospital of Liaoning University of Traditional Chinese Medicine, Shenyang, Liaoning, China

**Keywords:** central sensitization, neuro-immune axis, NLRP3 inflammasome, obesity-induced inflammation, specialized pro-resolving mediators, trained immunity

## Abstract

Obesity and chronic pain co-occur at high rates, yet the mechanistic logic that connects metabolic excess to persistent nociception remains fragmented across separate literatures. This review integrates these literatures around a single guiding question: how does chronic energy overload become a sensitized nervous system that no longer self-terminates? We propose a Metabolic–Immune–Neural (MIN) triangle hypothesis and trace a four-step causal chain. First, adipose tissue remodeling—hypertrophy, hypoxia, ER stress, lipid spillover, and metabolic endotoxemia—generates the molecular precursors of inflammation. Second, immune-cell remodeling and immunometabolic reprogramming amplify these signals, and trained immunity inscribes an epigenetic memory in myeloid cells and adipocytes that survives weight loss. Third, adipocyte–macrophage positive feedback loops, failed biosynthesis of specialized pro-resolving mediators (SPMs), and defective efferocytosis prevent resolution, so systemic low-grade chronic inflammation persists. Fourth, these signals converge on a three-tier sensitization cascade—peripheral (DRG neuro-immune unit), spinal (microglial–astrocytic disinhibition and NMDA-LTP), and supraspinal (descending-control imbalance with vagal–HPA brake failure)—producing four clinically tractable phenotypes: metabolic osteoarthritis, diabetic small-fiber neuropathy, mixed-mechanism low back pain, and sex-dimorphic pain. This framework supports a precision-medicine strategy in which NLRP3 and Na_v_1.8 inhibitors, SPM analogs, GLP-1 receptor agonists, vagus nerve stimulation, and epigenetic modulators can be combined according to a patient’s metabolic–immune phenotype. We position obesity-related chronic pain not as a universal “terminal complication” of metabolic disease, but as a frequent and mechanistically explicable consequence of unresolved metabolic inflammation—one in which the depth of sensitization, rather than weight loss alone, defines therapeutic outcome.

## Graphical Abstract

**Figure:**
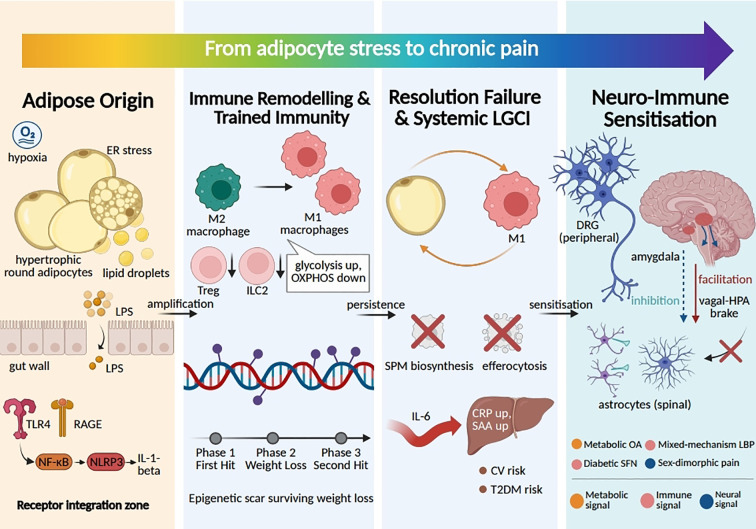
A four-panel schematic that walks the central argument of the review in one read. Panel 1 (Adipose Origin): hypertrophic adipocytes, hypoxia, ER stress, lipid spillover, and gut-derived LPS converge on TLR4/RAGE → NF-κB → NLRP3 → IL-1β. Panel 2 (Immune Remodeling and Trained Immunity): M2→M1 polarization, Treg/ILC2 collapse, immunometabolic rewiring, and the epigenetic scar that survives weight loss. Panel 3 (Resolution Failure and Systemic LGCI): adipocyte–macrophage positive feedback, defective SPM biosynthesis, impaired efferocytosis, and IL-6 driving the hepatic CRP/SAA response. Panel 4 (Neuro-Immune Sensitization): peripheral DRG → spinal microglia/astrocyte → supraspinal descending-control and vagal–HPA brake failure, feeding the four representative clinical pain phenotypes. Arrows trace one continuous causal chain from adipocyte stress to chronic pain; color coding distinguishes metabolic, immune, and neural signals. ER, endoplasmic reticulum; LPS, lipopolysaccharide; TLR4, Toll-like receptor 4; RAGE, receptor for advanced glycation end products; NF-κB, nuclear factor kappa B; NLRP3, NLR family pyrin domain containing 3; IL-1β/6, interleukin-1β/6; M1/M2, M1/M2 macrophage; Treg, regulatory T cell; ILC2, type 2 innate lymphoid cell; SPM, specialized pro-resolving mediator; LGCI, systemic low-grade chronic inflammation; CRP, C-reactive protein; SAA, serum amyloid A; DRG, dorsal root ganglion; HPA, hypothalamic–pituitary–adrenal.

## 1 Introduction

### 1.1 Global epidemiology of obesity

Obesity has become one of the most challenging non-communicable diseases of the 21st century. A pooled analysis of 3–663 population-representative surveys covering 222 million children, adolescents, and adults showed that the global obese population exceeded one billion in 2022, with a continued rise in most countries since 1990 (1). Under current trends, an estimated 3.8 billion adults are projected to be overweight or obese by 2050, with the fastest increases concentrated in low- and lower-middle-income countries (2). Chronic low-grade inflammation of adipose tissue is a core pathological feature of obesity and is now considered deeply involved in obesity-related metabolic dysfunction and multi-organ comorbidities (3).

### 1.2 Comorbidity of chronic pain with obesity and metabolic syndrome

The prevalence of chronic pain is markedly higher in people with obesity than in the general population. A systematic review and meta-analysis demonstrated that adults with BMI ≥ 30 kg/m² report greater pain intensity than normal-weight controls, whereas the overweight group (BMI 25–30 kg/m²) does not differ significantly from controls, suggesting a clinical “BMI threshold effect” (4). Imaging evidence from 32–409 abdominal MRI scans in UK Biobank further supports a dose–response relationship between both visceral and subcutaneous adipose tissue and multisite and widespread chronic pain, with stronger effect sizes in women (5). A cross-sectional analysis of the All of Us cohort showed that the additive predictive value of waist/hip circumference together with inflammatory markers (CRP, IL-6, leptin) for chronic pain was significant only in women, indicating sexual dimorphism in adipose–inflammation interactions (6).

Evidence also links metabolic syndrome (MetS) to chronic pain. A scoping review of 28 observational studies found that MetS is enriched among individuals with migraine, spinal pain, fibromyalgia, and general chronic pain, and that the two conditions are bidirectionally associated (7). The STEP 9 phase III randomized controlled trial provided direct clinical evidence: in patients with concurrent obesity and knee osteoarthritis, 68-week semaglutide treatment produced concurrent and significant improvements in body weight and knee WOMAC scores (8). These findings support the clinical tractability of a “weight loss–anti-inflammation–analgesia” axis.

### 1.3 The “metabolism–immunity–neural” triangle hypothesis

Integrating the above evidence, we propose a Metabolic–Immune–Neural (MIN) triangle hypothesis. Its central tenet is that chronic energy overload is transmitted to the nervous system via immune dysregulation, ultimately driving peripheral and central sensitization (9). Within this framework, metabolic abnormalities and inflammation interact through systemic mediators and the neural microenvironment, constituting a key mechanistic basis for metabolic pain phenotypes such as osteoarthritis (10).

The hypothesis emphasizes three pivotal nodes. First, the NLRP3 inflammasome can be activated by lipotoxicity-related damage-associated molecular patterns (DAMPs), promoting caspase-1–mediated maturation of IL-1β and IL-18; it is highly activated in metabolic disorders such as obesity and insulin resistance (11). The NLRP3–IL-1β axis also participates in peripheral and central sensitization in neuropathic and inflammatory pain, providing a mechanistic link between metabolic disturbances such as diabetes and chronic pain (12). Second, diet-induced obesity imprints persistent chromatin remodeling and pro-inflammatory memory in myeloid cells that endures even after body weight and metabolic indices have normalized, resulting in exaggerated inflammatory responses to subsequent stimuli (13); adipose tissue itself retains an analogous epigenetic memory, predisposing individuals to renewed metabolic dysfunction upon weight regain (14). Third, deficient biosynthesis or signaling of specialized pro-resolving mediators (SPMs) — including resolvins, protectins, and maresins — impairs active resolution of acute inflammation, allowing persistent low-grade inflammation to drive the chronification of pain (15).

Building on this framework, this review systematically examines the multisystem mechanisms linking adipose tissue to central sensitization, discusses the biological basis of representative clinical pain phenotypes (knee osteoarthritis, diabetic small-fiber neuropathy, and chronic low back pain) and their sex differences, and outlines therapeutic prospects targeting NLRP3, SPMs, GLP-1, and epigenetic modifiers, with the goal of informing precision interventions for the obesity–pain comorbidity.

## 2 Adipose tissue origin of inflammation

**Figure 1:**
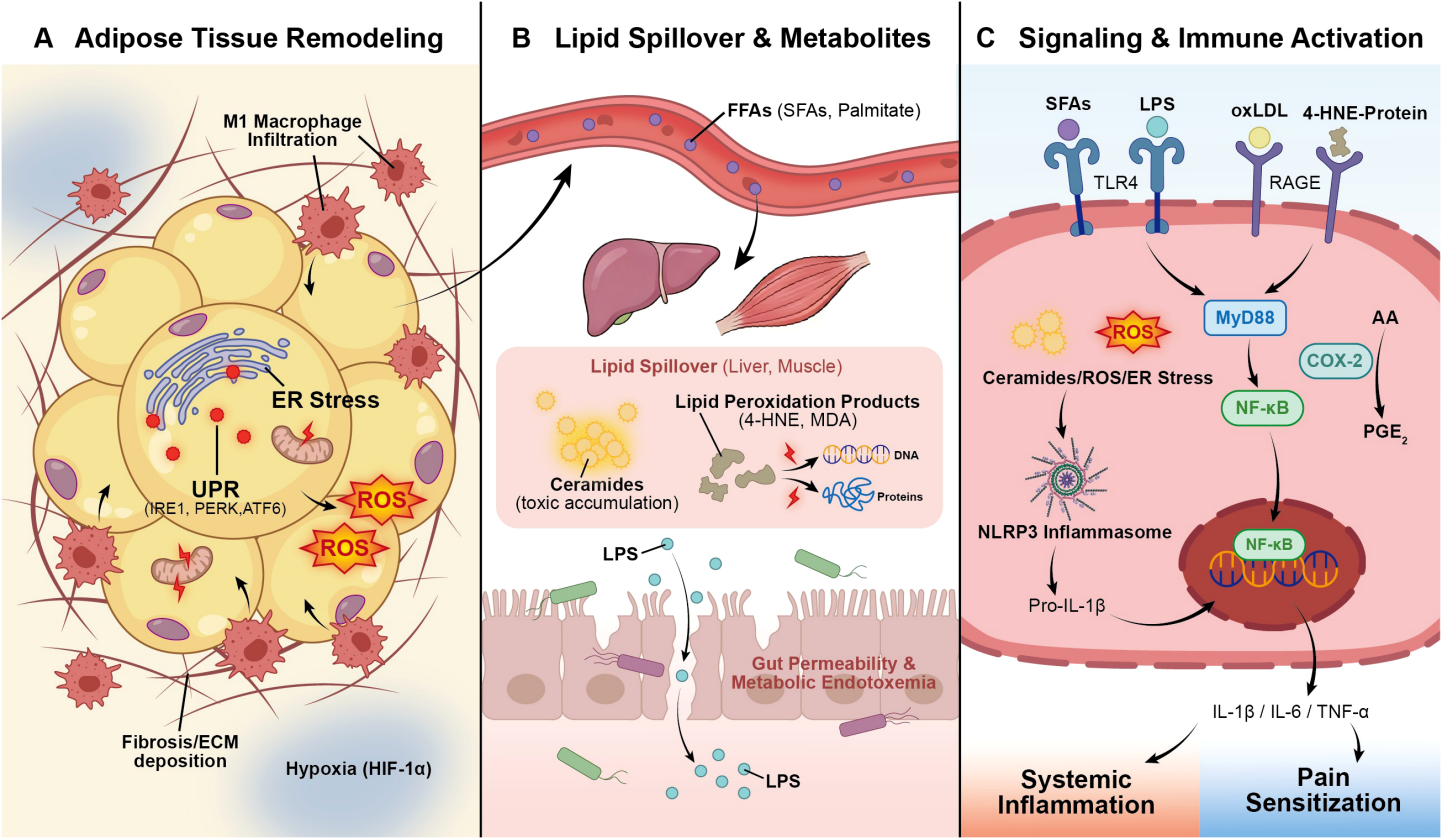
Adipose tissue remodeling, lipid spillover, and immune signaling activation under obesity. **(A)** Adipose Tissue Remodeling. Under chronic energy overload, adipocyte hypertrophy is accompanied by M1 macrophage infiltration, mitochondrial dysfunction, and elevated reactive oxygen species (ROS). Endoplasmic reticulum (ER) stress activates the unfolded protein response (UPR) through three branches — IRE1, PERK, and ATF6 — while localized hypoxia induces hypoxia-inducible factor 1-alpha (HIF-1α) stabilization. These events converge to drive extracellular matrix (ECM) deposition and adipose tissue fibrosis. **(B)** Lipid Spillover and Metabolites. Once adipocyte storage capacity is saturated, free fatty acids (FFAs) — predominantly saturated fatty acids (SFAs) and palmitate — undergo ectopic deposition in the liver and skeletal muscle. Ceramides accumulate to toxic levels, and lipid peroxidation products 4-hydroxynonenal (4-HNE) and malondialdehyde (MDA) form covalent adducts with DNA and proteins. In parallel, disruption of the intestinal barrier permits lipopolysaccharide (LPS) translocation into the systemic circulation, producing metabolic endotoxemia that amplifies whole-body inflammation. **(C)** Signaling and Immune Activation. SFAs and LPS engage the Toll-like receptor 4 (TLR4)–MyD88 pathway, while oxidized low-density lipoprotein (oxLDL) and 4-HNE–protein adducts activate the receptor for advanced glycation end products (RAGE); both converge on nuclear factor kappa B (NF-κB). Ceramides, ROS, and ER stress jointly trigger NLRP3 inflammasome assembly and processing of pro-IL-1β into mature interleukin-1 beta (IL-1β). Arachidonic acid (AA) is metabolized by cyclooxygenase-2 (COX-2) to prostaglandin E_2_ (PGE_2_). The net output of IL-1β, interleukin-6 (IL-6), and tumor necrosis factor-alpha (TNF-α) drives systemic inflammation and peripheral pain sensitization. Arrow directionality reflects experimentally supported causal or signaling relationships rather than purely visual flow; bidirectional arrows are used only where reciprocal regulation has been demonstrated. ROS, reactive oxygen species; ER, endoplasmic reticulum; UPR, unfolded protein response; IRE1, inositol-requiring enzyme 1; PERK, protein kinase R-like endoplasmic reticulum kinase; ATF6, activating transcription factor 6; HIF-1α, hypoxia-inducible factor 1-alpha; ECM, extracellular matrix; FFAs, free fatty acids; SFAs, saturated fatty acids; 4-HNE, 4-hydroxynonenal; MDA, malondialdehyde; LPS, lipopolysaccharide; TLR4, Toll-like receptor 4; MyD88, myeloid differentiation primary response 88; oxLDL, oxidized low-density lipoprotein; RAGE, receptor for advanced glycation end products; NF-κB, nuclear factor kappa B; NLRP3, NLR family pyrin domain containing 3; IL-1β/6, interleukin-1β/6; AA, arachidonic acid; COX-2, cyclooxygenase-2; PGE_2_, prostaglandin E_2_; TNF-α, tumor necrosis factor-alpha.

The molecular starting point of obesity–pain comorbidity resides within adipose tissue (**Figure 1**). To make the mechanistic connections explicit, we organize this section into three sequential blocks that follow the path of a single inflammatory signal: (i) Tissue-level remodeling (Sections 2.1 hypertrophy/hypoxia/ER stress; 2.2 macrophage infiltration and CLS; 2.3 ECM stiffening) generates the cellular *substrate* for inflammation; (ii) Lipid spillover and barrier failure (Sections 2.4 ceramides and lipid peroxidation; 2.5 gut barrier and metabolic endotoxemia) supply the *ligands and DAMPs* that escape from this remodeled tissue; (iii) Receptor-level integration (Sections 2.6 TLR4/RAGE→NF-κB; 2.7 NLRP3→IL-1β) translates these ligands into transcriptional and post-translational inflammatory output that ultimately sensitizes nociceptors. Each subsection ends by handing off its molecular product to the next, so that adipocyte stress, lipid metabolites, and pattern-recognition signaling are read as one continuous cascade rather than three parallel descriptions. Together, these events converge to form the molecular basis of systemic inflammation and pain sensitization.

### 2.1 Adipocyte hypertrophy, hypoxia and endoplasmic reticulum stress

Under chronic energy overload, adipose tissue expands primarily through adipocyte hypertrophy (**Figure 1A**). As cell diameter increases, the diffusion limit of oxygen is exceeded and diffuse tissue hypoxia develops. HIF-1α stabilization in hypoxic adipocytes initiates pro-fibrotic and pro-inflammatory transcriptional programs and drives adipose tissue toward a state of failed lipid storage (3).

Nutrient overload and saturated fatty acid exposure increase the protein-folding burden of the endoplasmic reticulum. Under sustained stress, the unfolded protein response (UPR) shifts toward a pro-inflammatory mode and amplifies inflammatory signaling through the JNK–IKK–NF-κB cascade (16). The UPR also acts as a central hub linking adipose tissue dysfunction to metabolic inflammation and induces adipocyte apoptosis via CHOP/ATF4 (17). Mitochondrial oxidative phosphorylation is impaired and generates excess reactive oxygen species (ROS), which in turn stabilize HIF-1α and reinforce the hypoxia–ROS–inflammation positive feedback loop (3).

The resulting hypoxic, ER-stressed, and ROS-rich microenvironment is the cellular substrate on which the immune-cell infiltration described next is recruited and reprogrammed.

### 2.2 Immune cell infiltration, M1 polarization and crown-like structure formation

In obese adipose tissue, macrophages shift from an anti-inflammatory M2 phenotype to a pro-inflammatory M1 phenotype and aggregate around dead or apoptotic adipocytes to form characteristic crown-like structures (CLS). The density of CLS correlates positively with the intensity of adipose tissue inflammation, with insulin resistance and with the risk of type 2 diabetes mellitus (T2DM) (18).

Single-cell sequencing has identified novel macrophage subsets, including lipid-associated macrophages (LAMs), metabolically activated macrophages (MMe) and CD9^+^ macrophages, which reside primarily within CLS and participate in lipid droplet uptake and matrix remodeling (19). Adaptive immunity is also reshaped: in obese visceral adipose tissue, CD8^+^ effector memory T cells and Th1 cells expand, whereas regulatory T cells decrease (20).

A history of obesity imprints an inflammatory memory on myeloid cells through epigenetic modifications and allows subsequent stimuli to elicit exaggerated inflammatory responses even after body weight normalizes (13). Human and murine adipose tissues themselves retain epigenetic marks of prior obesity, providing a molecular explanation for the metabolic memory observed after weight loss (14). As these studies directly establish persistent epigenetic and inflammatory memory in murine myeloid cells and adipose tissue after weight loss; their extension to the persistence of clinical chronic pain after metabolic correction is mechanistically plausible but is framed here as a working hypothesis pending direct pain-related human evidence (see expanded discussion in Section 3.3 and Section 8.3).

### 2.3 ECM deposition, fibrosis and increased matrix stiffness

Healthy adipose tissue expansion depends on coordinated remodeling of the extracellular matrix (ECM). In obesity, hypoxia–HIF-1α signaling and the TGF-β/SMAD cascade jointly drive excessive collagen deposition. Lysyl oxidase (LOX)–mediated irreversible cross-linking further increases matrix stiffness, and endotrophin released from cleaved type VI collagen α3 acts as a central pro-fibrotic and pro-inflammatory adipokine (21). Mechanical–biochemical feedback between adipocytes and the matrix restricts the recruitment of new adipocytes and thereby aggravates lipid spillover and ectopic deposition (22). Low-molecular-weight hyaluronan fragments generated during matrix turnover act as DAMPs and activate innate immune cells via TLR2/4, further amplifying local inflammation (23).

Matrix-imposed restriction of further adipocyte recruitment is the structural reason why excess lipid can no longer be safely stored, and it therefore sets the stage for the lipid spillover and ectopic deposition addressed next.

### 2.4 Lipid spillover, ceramides and lipid peroxidation products

Once the storage capacity of adipocytes is exceeded, free fatty acids — particularly saturated fatty acids and palmitate — spill over into the liver, skeletal muscle, pancreas and peripheral nervous system, causing ectopic lipid deposition and lipotoxicity (**Figure 1B**) (10).

Palmitoyl-CoA enters the *de novo* ceramide synthesis pathway through serine palmitoyltransferase. C16:0, C18:0 and C24:1 ceramides accumulate within plasma membranes, mitochondria and lysosomes, suppressing Akt phosphorylation and impairing mitochondrial function (24). Plasma ceramide levels have been validated in prospective cohorts as independent biomarkers of cardiovascular events, T2DM and metabolic syndrome (25).

ROS attack polyunsaturated fatty acids to generate the lipid peroxidation products 4-HNE and MDA. These reactive species form covalent adducts with DNA and proteins, suppress adiponectin secretion, activate hormone-sensitive lipase (HSL) and amplify NF-κB–driven transcription (26).

### 2.5 Disruption of the intestinal barrier and metabolic endotoxemia

High-fat, high-glucose diets cause intestinal dysbiosis, characterized by an elevated Firmicutes-to-Bacteroidetes ratio and reduced abundance of *Akkermansia muciniphila*. The same diets downregulate tight-junction proteins such as occludin and ZO-1 and render the intestinal barrier permeable (27). Lipopolysaccharide (LPS) subsequently enters the systemic circulation via the portal vein and chylomicrons and produces a sustained subclinical elevation known as metabolic endotoxemia (28). Circulating LPS then activates adipose tissue macrophages and hepatic Kupffer cells through the CD14–MD2–TLR4 complex, serving as the central environmental signal that links gut dysbiosis with inflammation in adipose tissue and the liver (28). Together with the ceramides and 4-HNE/MDA adducts generated within adipose tissue itself (Section 2.4), gut-derived LPS forms the second class of ligands feeding into the receptor-level integration described next.

### 2.6 TLR4 and RAGE pathways activate NF-κB and the COX-2/PGE2 axis

Two classes of pattern recognition receptors form the signaling hub of adipose tissue inflammation (**Figure 1C**).

Saturated fatty acids remodel membrane lipid composition and lipid raft assembly and thereby lower the activation threshold of the TLR4–MD2 complex to LPS. MyD88 then recruits IRAK4/1–TRAF6, ultimately activating IκB kinase and NF-κB (29). This pathway produces a dual-signal effect in adipose tissue exposed to subclinical endotoxemia and elevated circulating FFAs, markedly aggravating inflammation and insulin resistance.

RAGE recognizes metabolic DAMPs such as AGEs, HMGB1, S100 proteins and 4-HNE–protein adducts. Its expression is upregulated in obesity and cardiovascular disease, making it a central inflammatory pathway linking obesity to cardiovascular complications (30). Within adipose tissue, RAGE activation directly affects adipocyte differentiation, lipid metabolism and adipokine secretion (31).

Both pathways converge on NF-κB and drive the transcription of TNF-α, IL-6, MCP-1, COX-2 and iNOS. COX-2 catalyzes the conversion of arachidonic acid to PGE2, which sensitizes peripheral nociceptors via EP1/EP4 receptors and functions as a key mediator linking adipose tissue inflammation to peripheral pain sensitization (32). NF-κB activation provides only the *priming* signal; the *activation* signal that converts pro-IL-1β into mature IL-1β is delivered by the NLRP3 inflammasome described below.

### 2.7 NLRP3 inflammasome and IL-1β maturation: from systemic inflammation to pain sensitization

NF-κB–driven priming signals upregulate the transcription of NLRP3, pro-IL-1β and pro-IL-18. SFA metabolites, cholesterol crystals, uric acid crystals, mitochondrial ROS and lysosomal rupture then provide the activation signal that triggers NLRP3 oligomerization, ASC recruitment and Caspase-1 autocleavage, leading to the maturation of IL-1β, IL-18 and GSDMD (33). In adipose tissue, hepatic Kupffer cells and pancreatic β cells, NLRP3 activation correlates closely with the risk of T2DM, NAFLD/NASH and cardiovascular events; the IL-1 receptor antagonist anakinra, canakinumab and the oral NLRP3 inhibitor dapansutrile have shown glucose-lowering and anti-inflammatory benefits in multiple clinical trials (33). These trials evaluated metabolic and cardiovascular endpoints; the analgesic efficacy of NLRP3-pathway or IL-1-blocking agents in obesity-related chronic pain has not been established in dedicated pain RCTs and is currently inferred from mechanistic and metabolic-disease data rather than from direct pain-outcome evidence.

The NLRP3 inflammasome is also activated in spinal dorsal horn microglia and dorsal root ganglion neurons, and the release of IL-1β and IL-18 is a key driver of both inflammatory and neuropathic pain (12). IL-1β, IL-6 and TNF-α act on dorsal root ganglion neurons to upregulate TRPV1 expression and phosphorylation, lowering the activation threshold of peripheral nociceptors to thermal and chemical stimuli (34). The combined effect of peripheral sensitization and the central action of systemic inflammatory mediators constitutes the neuroimmune basis of the exaggerated pain responses observed in obese individuals to routine stimuli (9). It should be emphasized that this output represents sustained low-grade IL-1β/TNF-α release rather than an acute cytokine-storm response, distinguishing obesity-related chronic inflammation from sepsis-like physiology.

The continuous signaling chain from adipocyte stress to pain sensitization represents the core mechanism of the metabolic–immune–neural axis at the adipose tissue level. It also forms the shared molecular basis for the obesity-related comorbidities addressed in subsequent sections, including knee osteoarthritis, diabetic small-fiber neuropathy and chronic low back pain (35).

## 3 Immune cell remodeling and immunometabolism

**Figure 2:**
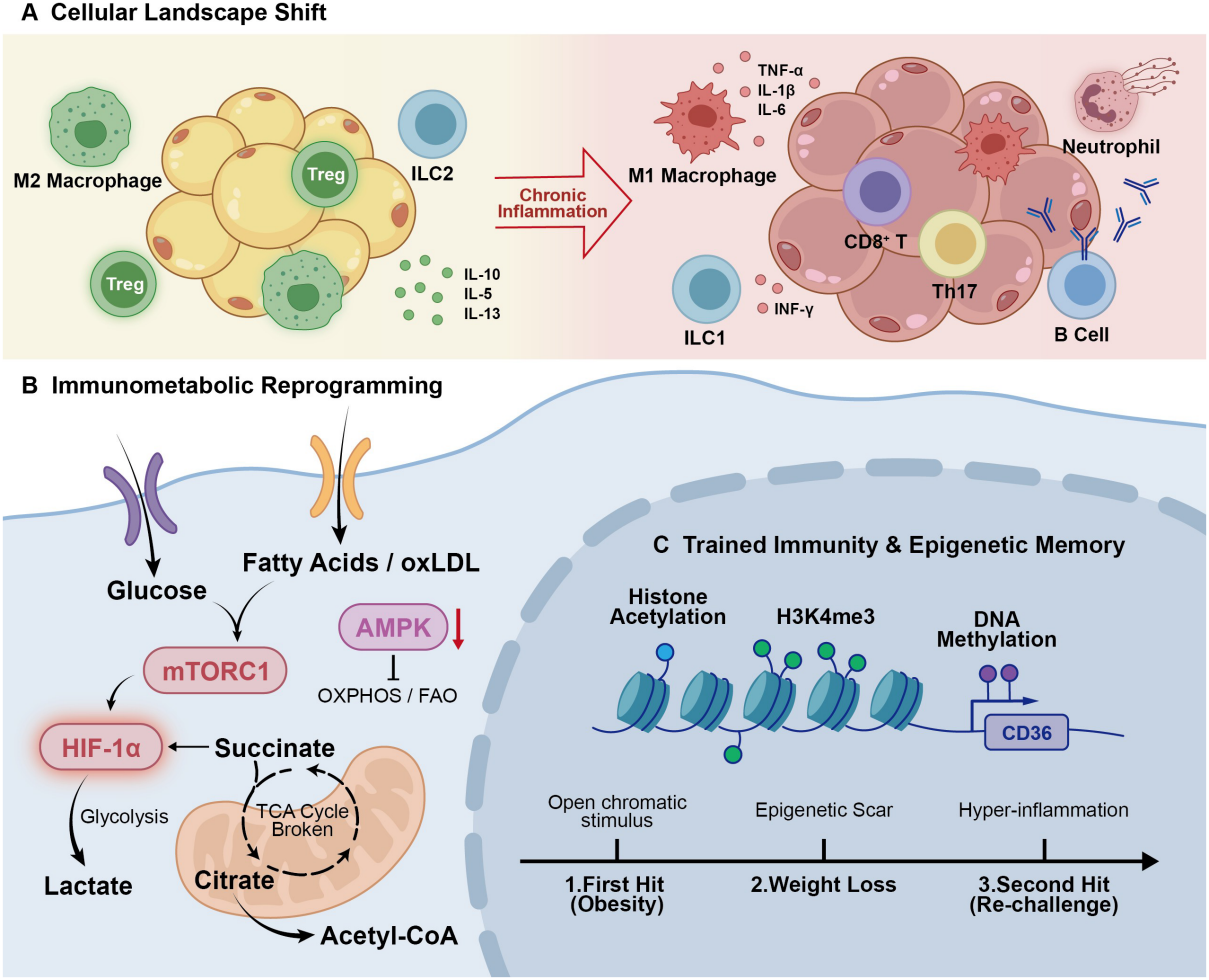
Immune cell landscape shift, immunometabolic reprogramming, and epigenetic memory in obese adipose tissue. **(A)** Cellular Landscape Shift. Healthy adipose tissue is dominated by M2 macrophages, regulatory T cells (Tregs), and type 2 innate lymphoid cells (ILC2s), which collectively secrete interleukin-10 (IL-10), interleukin-5 (IL-5), and interleukin-13 (IL-13) to maintain homeostasis. Under chronic obesity, the cellular landscape shifts toward M1 macrophage, CD8^+^ T cell, T helper 17 (Th17), ILC1, B cell, and neutrophil dominance, accompanied by abundant release of TNF-α, IL-1β, IL-6, and interferon-gamma (IFN-γ). **(B)** Immunometabolic Reprogramming. Immune cells transition from oxidative phosphorylation (OXPHOS) to aerobic glycolysis. Glucose and FAs/oxLDL uptake increase; mechanistic target of rapamycin complex 1 (mTORC1) is activated, AMP-activated protein kinase (AMPK) is suppressed, and OXPHOS and fatty acid oxidation (FAO) are downregulated. Tricarboxylic acid (TCA) cycle disruption causes succinate accumulation, which stabilizes HIF-1α and promotes glycolytic flux with lactate output. Citrate exits the mitochondria and is converted to cytosolic acetyl-coenzyme A (acetyl-CoA), supplying substrate for lipid biosynthesis and histone acetylation. **(C)** Trained Immunity and the Epigenetic Scar. A three-phase timeline depicts the persistence of inflammatory memory across weight loss. Phase 1 — First Hit (Obesity): upstream metabolic cues including oxLDL/CD36, hyperglycemia, saturated fatty acids, advanced glycation end products (AGEs) and ceramide-derived metabolic damage-associated molecular patterns (DAMPs) deposit activating histone marks (H3K4me3, H3K4me1, H3K27ac) at inflammatory gene loci, alter DNA methylation at CD36 and NF-κB-pathway promoters, and reshape chromatin accessibility in both bone-marrow CD34^+^ hematopoietic stem/progenitor cells (HSPCs; central training) and tissue-resident macrophages (peripheral training). Phase 2 — Weight Loss: body weight and circulating metabolic indices normalize, but the histone-methylation, histone-acetylation, DNA-methylation and chromatin-accessibility marks above persist, constituting the “epigenetic scar.” Phase 3 — Second Hit (Re-challenge): a subsequent metabolic or inflammatory stimulus rapidly reactivates the scarred loci, producing supraphysiological IL-1β, TNF-α and IL-6 output that drives renewed peripheral and central pain sensitization. Persistent adipocyte transcriptomic memory parallels the immune scar on the adipose side, so the framework operates simultaneously on the immune and adipose limbs of the metabolic–immune axis. Arrow directionality reflects experimentally supported causal or signaling relationships rather than purely visual flow; bidirectional arrows are used only where reciprocal regulation has been demonstrated. ATM, adipose tissue macrophage; Treg, regulatory T cell; ILC1/2, type 1/2 innate lymphoid cell; Th17, T helper 17 cell; IL-5/10/13, interleukin-5/10/13; IL-1β/6, interleukin-1β/6; TNF-α, tumor necrosis factor-alpha; IFN-γ, interferon-gamma; OXPHOS, oxidative phosphorylation; FAs, fatty acids; oxLDL, oxidized low-density lipoprotein; mTORC1, mechanistic target of rapamycin complex 1; AMPK, AMP-activated protein kinase; FAO, fatty acid oxidation; TCA, tricarboxylic acid; HIF-1α, hypoxia-inducible factor 1-alpha; acetyl-CoA, acetyl-coenzyme A; SFAs, saturated fatty acids; NF-κB, nuclear factor kappa B; H3K4me3, histone 3 lysine 4 trimethylation; CD36, fatty acid translocase/oxLDL scavenger receptor; AGEs, advanced glycation end products; DAMPs, damage-associated molecular patterns; H3K4me1, histone 3 lysine 4 monomethylation; H3K27ac, histone 3 lysine 27 acetylation; HSPC, hematopoietic stem/progenitor cell.

The key amplification node of obesity–pain comorbidity lies in the systemic reprogramming of the immune–metabolic axis within adipose tissue (**Figure 2**). This reprogramming unfolds at three levels: an overall shift in the cellular landscape from anti-inflammatory homeostasis toward chronic inflammation; a simultaneous reprogramming of carbohydrate–lipid metabolism and mitochondrial function within individual immune cells; and the establishment of trained immunity, through which inflammation forms an epigenetic “imprint” that can be reignited upon re-challenge even after weight loss. Following this structure, the present section addresses M2→M1 polarization with adaptive immune drift, immunometabolic reprogramming driven by glucose and FA/oxLDL, and trained immunity together with the epigenetic scar mediated by histone modifications and DNA methylation. These three layers of reprogramming jointly provide the immunological explanation for the “weight loss does not abolish pain” phenomenon, and they form the central mechanistic relay between the adipose-tissue origin of inflammation (**Figure 1**) and the target-organ injuries discussed in subsequent sections.

### 3.1 Shift of the cellular landscape: from anti-inflammatory homeostasis to pro-inflammatory remodeling

In healthy adipose tissue, immune cells form a network anchored on anti-inflammation and homeostasis. M2 adipose tissue macrophages (ATMs) predominate and use nutritional and metabolic cues to establish bidirectional immune–metabolic coupling with adipocytes, sustaining local insulin sensitivity (36). Tregs, ILC2s, and Th2 cells together constitute a type 2 immune axis that stabilizes M2 polarization and lipid storage through IL-5, IL-10, and IL-13 (37). VAT-resident Tregs rely on the PPARγ program and TCR clonal expansion, and they display unique transcriptomic and TCR repertoires that evolve dynamically with age and metabolic load (38).

Chronic energy overload disrupts this homeostasis (**Figure 2A**). ATMs shift from M2 toward M1, and the macrophage and T cell networks are reshaped in concert (19). In obese VAT, distinct macrophage subsets are recognized, including lipid-associated macrophages (LAMs), metabolically activated macrophages (MMe), and CD9^+^ macrophages. These cells do not conform fully to the classical M1 definition, yet they similarly express high levels of pro-inflammatory and lipid-handling genes (19). Under obesity, ATMs adopt a unique “double-high” metabolic state characterized by simultaneously elevated glycolysis and OXPHOS, distinguishing them from classical LPS-activated M1 macrophages (39).

Adaptive immunity also tilts toward inflammation. CD8^+^ effector memory T cells and Th1 cells in obese VAT expand markedly with rising BMI, and IFN-γ becomes the central signal driving M1 polarization (40). VAT Treg numbers and function decline significantly in obesity, with elevated PD-1 expression and impaired clonal expansion (41). Single-cell sequencing has resolved the dynamic differentiation trajectories of VAT Treg subsets across diet and age, revealing heterogeneity far greater than previously appreciated (38). This heterogeneity is shaped by transcription factors such as PPARγ and the estrogen receptor, together with sex hormones, offering a new explanation for the sex-dependent differences in inflammatory phenotype among obese individuals (42). VAT Tregs—particularly the ST2hi subset—depend on SREBP2-mediated cholesterol biosynthesis to maintain homeostatic expansion; obesity disrupts Treg cholesterol homeostasis and selectively depletes this subset, exacerbating VAT inflammation and insulin resistance (43). Dendritic cells in obese VAT upregulate the maturation markers MHC-II, CD86, and PD-L1 and suppress Treg differentiation and proliferation through downregulation of the IL-33 axis, providing an upstream signal that biases the network toward inflammation (44).

Innate lymphoid cell “switching” is equally critical. ILC2 numbers and function are impaired in obese VAT, dampening their anti-inflammatory capacity, while ILC1 numbers rise in parallel and drive M1 polarization and adipose tissue fibrosis through IFN-γ (45).

B cells and neutrophils also participate in obese VAT inflammation. B2 cells expand in obesity and aggravate tissue inflammation by secreting pathogenic antibodies such as IgG2c (20). Neutrophils are the earliest innate immune cells to infiltrate adipose tissue; they amplify local inflammation and directly impair insulin signaling through neutrophil elastase, IL-1β, and myeloperoxidase. The neutrophil-to-lymphocyte ratio (NLR) has accordingly become a clinically measurable indicator of systemic inflammation in obesity (46).

Together, this cellular-landscape shift can be summarized at the molecular level as collapse of the IL-10/IL-5/IL-13 homeostatic network and emergence of the TNF-α/IL-1β/IL-6/IFN-γ pro-inflammatory network, after which immune–metabolic coupling proceeds to the next layer of reprogramming (47).

### 3.2 Immunometabolic reprogramming: the glycolysis–HIF-1α axis, TCA cycle break, and AMPK suppression

The polarization states of macrophages and T cells are tightly coupled to their metabolic pathways, a phenomenon termed immunometabolism. With ATMs at the center, the intracellular metabolic axes undergo coordinated reprogramming in obesity (**Figure 2B**): glucose enters through GLUT1/3 and activates mTORC1, which stabilizes HIF-1α to drive glycolytic flux and lactate output; fatty acids and oxidized LDL (oxLDL) enter through membrane transporters; AMPK activity is downregulated, suppressing OXPHOS and FAO; and the TCA cycle breaks at multiple nodes, so that succinate, citrate, acetyl-CoA, and itaconate are elevated from metabolic by-products to signaling molecules (47).

#### 3.2.1 The glucose–mTORC1–HIF-1α–glycolysis axis

Obesity-associated hormones and nutrient signals activate mTORC1 via the PI3K–AKT–mTORC1 cascade. mTORC1 stabilizes HIF-1α and initiates transcription of glycolytic enzymes and glucose transporters, driving the differentiation of M1 macrophages and effector T cells (47). In obese VAT, ATMs show increased nuclear translocation of HIF-1α, a phenomenon induced by saturated fatty acids such as palmitate under both hypoxic and non-hypoxic conditions; myeloid-specific deletion of HIF-1α markedly reduces ATM accumulation and IL-1β production in adipose tissue under high-fat diet, confirming HIF-1α as a central node of the pro-inflammatory ATM phenotype in obesity (48). As the terminal product of glycolysis, lactate further stabilizes HIF-1α by inhibiting PHD2, generating a lactate–HIF-1α–glycolysis positive feedback loop.

#### 3.2.2 TCA cycle break and the signaling roles of succinate and itaconate

M1 activation causes a critical TCA cycle break at the SDH node. Succinate accumulates in the mitochondrial matrix and spills into the cytosol, where it stabilizes HIF-1α through PHD inhibition and amplifies IL-1β signaling via the SUCNR1 receptor (49). Serum succinate is markedly elevated and correlates positively with IL-1β in patients with coronary artery disease, T2DM, and obesity, making it a measurable signaling molecule of metabolic inflammation (49). Citrate escapes the TCA cycle and is converted to acetyl-CoA by ATP citrate lyase, providing the substrate for histone acetylation and thereby linking metabolism to epigenetics.

Itaconate is another key node of obesity-associated inflammatory metabolism. Within M1 macrophages, IRG1/ACOD1 catalyzes the conversion of cis-aconitate to itaconate, which inhibits SDH to regulate succinate, activates the Nrf2 antioxidant pathway, and suppresses the NLRP3 inflammasome, forming an endogenous anti-inflammatory brake (50). In obesity, this anti-inflammatory capacity is overwhelmed by sustained pro-inflammatory signals, and the metabolic balance tilts toward inflammation.

#### 3.2.3 Fatty acid/oxLDL handling and the OXPHOS/FAO defect under AMPK suppression

In obesity, ATMs avidly take up fatty acids and oxLDL through scavenger receptors such as CD36. This both supplies lipid fuel and provides metabolic DAMPs that sustain low-grade inflammation. AMPK is the cellular energy sensor: under physiological conditions it promotes OXPHOS and FAO while limiting M1 polarization and NLRP3 activation, serving as an anti-inflammatory gatekeeper. Nutrient overload in obesity broadly suppresses AMPK activity, and the resulting decline in OXPHOS and FAO increases mitochondrial ROS production, which in turn stabilizes HIF-1α and amplifies NLRP3 activation (51). Dysregulation of this AMPK–FAO axis is also central to the excessive activation of monocytes and macrophages in inflammaging, an aging-related condition closely linked to obesity (51).

In summary, immunometabolic reprogramming is the engine that amplifies and entrenches the cellular-landscape shift. Overactivation of the glycolysis–HIF-1α axis, signaling metabolites derived from the broken TCA cycle, and suppression of the AMPK–FAO pathway act in concert to transform obese ATMs from passive responders into active inflammatory factories. The same reprogramming furnishes the substrates for the next-layer epigenetic imprint, because α-ketoglutarate, succinate, acetyl-CoA, and itaconate are direct cofactors or inhibitors of epigenetic modifying enzymes.

### 3.3 Trained immunity and the epigenetic scar: why inflammation can “reignite” after weight loss

The third layer follows a cautionary timeline (**Figure 2C**): a first hit (obesity), followed by weight loss that leaves an epigenetic scar, then a second hit (re-challenge) that culminates in hyper-inflammation. The concept of trained immunity has fundamentally revised the long-held view that innate immunity has no memory. Monocytes, macrophages, and myeloid progenitor cells imprint past stress into epigenetic memory through histone modifications, DNA methylation, and metabolic reprogramming, retaining an enhanced state of inflammatory responsiveness even after the original stimulus is removed (52). To make the progression from observed phenomenon to molecular implementation explicit, we present this account in five steps: the clinical observation that motivates the framework (Section 3.3.1), trained immunity as the mechanistic basis (Section 3.3.2), the upstream metabolic cues that induce and maintain the trained state (Section 3.3.3), the layered epigenetic mechanisms that implement it (Section 3.3.4), and its implications for the persistence of pain after weight loss (Section 3.3.5).

#### 3.3.1 Clinical observation: inflammatory memory persists after metabolic correction

A consistent clinical observation motivates this section: after diet-induced weight loss, pharmacotherapy, or bariatric surgery, body weight and metabolic indices may normalize, yet the pro-inflammatory capacity of myeloid cells and the inflammatory tone of adipose tissue do not (13, 14). Weight cycling further demonstrates that adipose tissue macrophages from previously obese mice mount stronger basal and stimulated cytokine responses, and multi-omics CITE-seq shows that the obesity-induced imprint on adipose immune cells worsens rather than resolves after weight regain (53, 54). This “metabolic memory” is the phenomenon that any mechanistic account of post-weight-loss residual inflammation must explain.

It should be noted, however, these studies establish persistent immune-cell memory in adipose tissue following weight cycling but do not by themselves establish persistent osteoarthritis pain, diabetic neuropathy, or chronic low back pain after weight loss; the inference from adipose-immune memory to persistent pain phenotypes is therefore framed here as a hypothesis to be tested rather than as a directly demonstrated link.

#### 3.3.2 Mechanistic basis: trained immunity and the epigenetic scar

As summarized in (**Figure 2C**), trained immunity provides the conceptual framework for these observations. Trained immunity provides the conceptual framework for these observations. Monocytes, tissue-resident macrophages, and bone-marrow myeloid progenitors record prior inflammatory exposures as durable changes in chromatin state, so that subsequent stimuli evoke disproportionately amplified responses (52). Within an obesity timeline, the first hit (diet-induced obesity) deposits this imprint, weight loss does not erase it, and a second hit (re-challenge, weight regain, or even routine inflammatory stress) reactivates it. The same logic applies to adipocytes themselves, which retain transcriptomic and DNA-methylation scars of prior obesity (14), so that the epigenetic scar operates simultaneously on the immune and adipose sides of the metabolic–immune axis.

#### 3.3.3 Upstream metabolic cues that induce and maintain the scar

The metabolic environment of obesity supplies the cues that establish trained immunity. OxLDL binding to CD36 induces a trained phenotype in monocytes after only brief stimulation, producing markedly enhanced cytokine output upon subsequent exposure to TLR2/4 ligands, and forms a self-reinforcing “uptake → training → stronger uptake” loop (55). Hyperglycemia similarly drives persistent inflammatory training: monocytes and macrophages exposed to diabetes or high glucose retain enhanced pro-inflammatory responses even after blood glucose normalizes, with sustained H3K4 methylation at NF-κB-pathway genes (56), while CD34^+^ hematopoietic stem cells from diabetic patients show persistent H3K4me1 enrichment, aberrant p65 NF-κB activation, and a senescence-associated secretory phenotype, linking central training to clinical metabolic memory (57). Beyond these representative inducers, metabolic damage-associated molecular patterns (DAMPs)—ceramides, 4-HNE/MDA adducts, saturated free fatty acids, AGEs, and HMGB1—act as repeated low-level priming events that maintain the trained state even when any single stimulus would be insufficient, positioning oxLDL/CD36 signaling and the broader metabolic-DAMP milieu as upstream triggers of the epigenetic mechanisms detailed in Section 3.3.4 rather than as epigenetic mechanisms themselves.

#### 3.3.4 Layered epigenetic mechanisms

The persistent imprint described above is implemented through four interacting layers of epigenetic regulation. (i) Histone methylation at H3K4me3 and H3K4me1 marks the promoters and enhancers of inflammatory genes for accelerated re-induction (52). (ii) Histone acetylation at H3K27ac, fueled by glycolysis- and broken-TCA-derived acetyl-CoA, increases enhancer accessibility and links immunometabolism directly to chromatin state (58). (iii) DNA methylation changes at CD36, NF-κB-pathway genes, and adipocyte identity loci stabilize the imprint across cell division (14, 57). (iv) Chromatin accessibility and non-coding RNAs, including changes at hematopoietic stem cell and tissue-macrophage enhancers, sustain training from bone-marrow progenitors through to peripheral effectors (52). Together, these four layers convert short-lived metabolic events into stable transcriptional bias, providing the molecular implementation of the epigenetic scar.

#### 3.3.5 Implications for the persistence of pain after weight loss

Because the epigenetic memory of the metabolic–immune axis is erased at neither the immune nor the adipose level, the upstream signals that drive pain sensitization—IL-1β, IL-6, TNF-α, PGE_2_—are expected to persist at low levels and to be rapidly amplified upon any secondary metabolic challenge. This pattern parallels the incomplete remission of comorbidities such as osteoarthritis pain, diabetic peripheral neuropathy, and chronic low back pain that is frequently observed after weight loss achieved through diet, pharmacotherapy, or bariatric surgery.

These studies directly establish persistent epigenetic and inflammatory memory after weight loss in murine myeloid cells and in adipose tissue, respectively; the connection between this molecular memory and the persistence of clinical chronic pain after metabolic correction is a mechanistically plausible extrapolation rather than a directly demonstrated link, and is therefore framed here as a working hypothesis that motivates dedicated human studies with pain endpoints (see Section 8.3).

The trained-immunity and epigenetic-scar mechanism (**Figure 2C**) integrates the cellular-landscape shift of Section 3.1 with the immunometabolic reprogramming of Section 3.2 along a temporal dimension, closing the inflammation–memory loop that subsequent sections translate into specific clinical pain phenotypes.

## 4 Positive feedback loops, resolution failure, and systemic low-grade chronic inflammation

**Figure 3:**
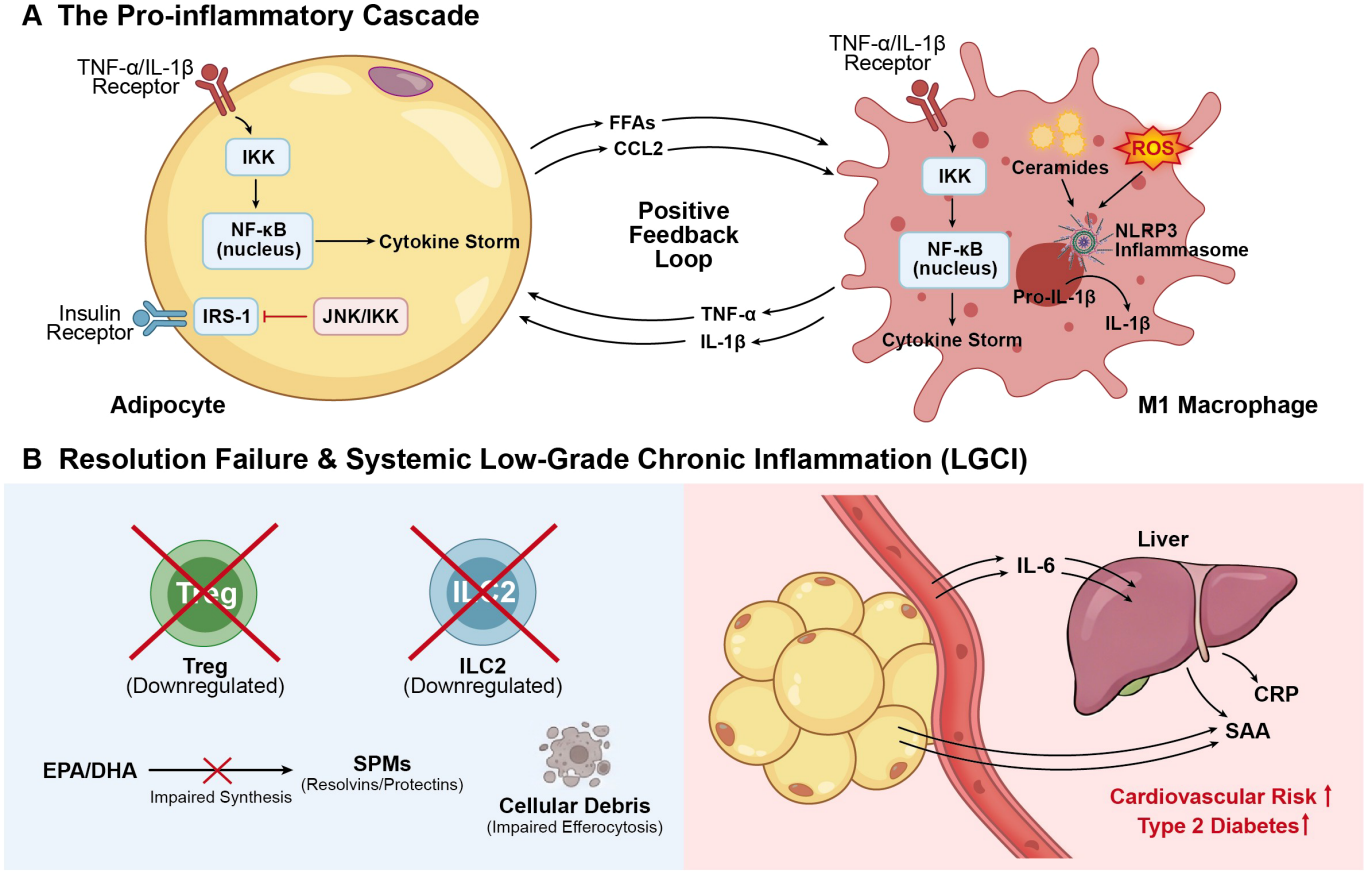
Pro-inflammatory positive feedback loop and resolution failure driving systemic low-grade chronic inflammation (LGCI). **(A)** The Pro-inflammatory Cascade. A positive feedback loop is established between adipocytes and M1 macrophages. Macrophage-derived TNF-α and IL-1β activate adipocyte TNF/IL-1 receptors, signaling through the IκB kinase (IKK)–NF-κB axis and driving cytokine production. Adipocytes in turn release FFAs and the chemokine C-C motif ligand 2 (CCL2), recruiting additional monocytes that differentiate into M1 macrophages. The c-Jun N-terminal kinase (JNK)/IKK cascade phosphorylates insulin receptor substrate-1 (IRS-1) at serine residues, suppressing insulin signaling and producing local insulin resistance. Within macrophages, ceramides and ROS synergistically activate the NLRP3 inflammasome, processing pro-IL-1β into mature IL-1β and amplifying the sustained low-grade IL-1β/TNF-α output characteristic of chronic metabolic inflammation, which is distinct from the acute cytokine-storm physiology of sepsis. **(B)** Resolution Failure and Systemic LGCI. Biosynthesis of specialized pro-resolving mediators (SPMs) — including resolvins and protectins — from eicosapentaenoic acid (EPA) and docosahexaenoic acid (DHA) is impaired. Treg and ILC2 populations are downregulated, and efferocytosis of apoptotic cells is defective, leading to cellular debris accumulation. IL-6 enters the portal circulation, stimulating hepatic synthesis of C-reactive protein (CRP) and serum amyloid A (SAA), which together elevate cardiovascular risk and the risk of type 2 diabetes mellitus (T2DM). Arrow directionality reflects experimentally supported causal or signaling relationships rather than purely visual flow; bidirectional arrows are used only where reciprocal regulation has been demonstrated. TNF-α, tumor necrosis factor-alpha; IL-1β/6, interleukin-1β/6; FFAs, free fatty acids; CCL2, C-C motif chemokine ligand 2; IKK, IκB kinase; NF-κB, nuclear factor kappa B; JNK, c-Jun N-terminal kinase; IRS-1, insulin receptor substrate-1; NLRP3, NLR family pyrin domain containing 3; ROS, reactive oxygen species; SPMs, specialized pro-resolving mediators; EPA, eicosapentaenoic acid; DHA, docosahexaenoic acid; Treg, regulatory T cell; ILC2, type 2 innate lymphoid cell; CRP, C-reactive protein; SAA, serum amyloid A; T2DM, type 2 diabetes mellitus; LGCI, systemic low-grade chronic inflammation.

Section 2 addressed the origin of inflammation in obese adipose tissue, and Section 3 described its amplification through immune-cell remodeling and immunometabolism. This section focuses on how inflammation becomes persistent along two axes. The first is the locking-in of a local positive feedback loop, in which adipocytes and M1 macrophages reinforce each other’s pro-inflammatory and insulin-resistant states through bidirectional paracrine signaling. The second is a systemic failure of resolution, in which the collapse of anti-inflammatory cell populations, defective production of specialized pro-resolving mediators (SPMs), and impaired efferocytosis prevent inflammation from resolving on its own. IL-6 then enters the circulation and activates the hepatic acute-phase response, raising the risk of cardiovascular events and type 2 diabetes (T2D). **Figure 3** integrates these local and systemic threads in panels 3A and 3B, respectively. This closed loop extends the principle that weight loss does not relieve pain from a single tissue to a body-wide chain of chronic disease, and it links to the target-organ injury discussed later.

### 4.1 The adipocyte–macrophage positive feedback loop

#### 4.1.1 The bidirectional paracrine loop: FFAs/CCL2 ↔ TNF-α/IL-1β

At the core of the loop is bidirectional paracrine communication between adipocytes and M1 macrophages: adipocytes export free fatty acids (FFAs) and CCL2, while macrophages export TNF-α and IL-1β in return (59). Hypertrophic adipocytes show enhanced basal lipolysis, and abundant saturated fatty acids reach neighboring macrophages through the interstitial space, acting as metabolic damage-associated molecular patterns (DAMPs) that activate TLR4 signaling (29). Adipocyte-derived CCL2 binds monocyte CCR2 and directs peripheral Ly6C^hi^ monocytes into adipose tissue, where they differentiate locally into M1 macrophages (60). Pharmacological blockade or genetic deletion of the CCL2/CCR2 axis markedly reduces adipose tissue macrophage numbers and improves insulin sensitivity (60). Macrophage-derived TNF-α acts back on adipocyte TNFR, activates the IKK→NF-κB cascade, and drives adipocytes to express more CCL2 and IL-6, thereby closing the loop (59). A macrophage–T-cell intercellular network further amplifies this loop and tightly couples local inflammation with systemic insulin resistance at the level of adipose tissue (18).

#### 4.1.2 IKK–NF-κB and cytokine amplification

NF-κB is the shared intracellular signaling hub of adipocytes and M1 macrophages. The IKK complex downstream of the TNF-α/IL-1β receptors phosphorylates IκBα, allowing NF-κB to translocate to the nucleus and initiate pro-inflammatory gene transcription (61). Nutrient and metabolic signals in the obese adipose microenvironment push macrophages toward the M1 phenotype, and these macrophages in turn reshape the metabolic fate of adjacent adipocytes, producing a coupled immune-shift–metabolic-switch (36). In obesity, adipose tissue macrophages (ATMs) adopt a dual-high metabolic state with simultaneously elevated glycolysis and oxidative phosphorylation (OXPHOS), yet their NF-κB-driven cytokine profile remains consistent with classical M1 (39). Nuclear translocation of HIF-1α in ATMs increases in obesity and substantially amplifies NLRP3 activation and IL-1β output, providing the metabolic basis for sustained IL-1β elevation in adipose tissue (48).

#### 4.1.3 Ceramide and ROS drive NLRP3 inflammasome activation in M1 macrophages

Within M1 macrophages, ceramide and reactive oxygen species (ROS) jointly activate the NLRP3 inflammasome and process pro-IL-1β into mature IL-1β (33). In obesity, abundant saturated fatty acids generate ceramide through the *de novo* synthesis pathway, and this ceramide serves as an endogenous danger signal that provides the second signal for NLRP3 activation (33). At the same time, mitochondrial dysfunction leads to ROS accumulation, which acts together with ceramide to trigger NLRP3 oligomerization, caspase-1 activation, and IL-1β secretion (33). IL-1β-neutralizing antibodies improve glycemic control in patients with T2D, confirming that this pathway is druggable (33). Adipose tissue IL-1β not only sustains local chronic inflammation but also acts on dorsal root ganglion nociceptors through the neuro-immune interface and lowers the pain threshold (12). NLRP3 activation also acts through key nociceptor ion channels such as TRPV1 to directly drive peripheral and central pain sensitization (34). Within the broader metabolic DAMP–neuro-immune framework, this axis serves as a central hub linking obesity to chronic pain (35). Two qualifications are worth flagging. First, the IL-1β neutralization evidence cited above (33) comes from trials with glycemic and metabolic endpoints, not from dedicated pain RCTs, so framing the NLRP3–IL-1β axis as an analgesic target in obesity-related chronic pain is for now a mechanism-based inference. Second, the IL-1β and TNF-α output described here is the sustained, low-grade kind seen in chronic metabolic disease — not the acute cytokine-storm physiology of sepsis, and the two should not be read interchangeably.

#### 4.1.4 JNK/IKK → IRS-1 serine phosphorylation → insulin resistance

On the adipocyte side, the loop has another key branch in which IRS-1, downstream of the insulin receptor, is inhibited by JNK/IKK. TNF-α, IL-1β, ceramide, and FFAs activate JNK and IKKβ, leading to excessive phosphorylation of IRS-1 at key sites such as Ser307, which suppresses its tyrosine phosphorylation and blocks PI3K–Akt signaling (61). This serine/tyrosine phosphorylation switch is the most direct molecular bridge between obesity-associated metabolic inflammation and insulin resistance (59). Insulin resistance abolishes the suppression of lipolysis, so FFAs are released continuously, which in turn intensifies pro-inflammatory stimulation of macrophages and allows the entire loop to be maintained over the long term (29, 59).

#### 4.1.5 Closing the loop

The processes described in 4.1.1–4.1.4 form not just four parallel pathways but a single closed circuit. Lipolysis sends lipid DAMPs to neighboring macrophages; macrophage NF-κB keeps adipocytes producing CCL2 and IL-6; NLRP3 matures the cytokine pool into IL-1β; IL-1β and TNF-α then phosphorylate IRS-1 at serine residues, which releases lipolysis from the brake of insulin signaling and restarts the cycle. The inputs into each node are redundant enough that removing any single stimulus does not switch the circuit off — which is precisely why this loop, once established locally in obese adipose tissue, becomes the seed for the systemic resolution failure addressed in Section 4.2.

### 4.2 Resolution failure: defective SPM synthesis and loss of immunoregulatory cells

Resolution failure can be dissected into three interconnected nodes—loss of anti-inflammatory regulatory cells, defective SPM synthesis, and impaired efferocytosis—which together strip inflammation of its capacity to terminate actively.

#### 4.2.1 Loss of Tregs and ILC2s: collapse of the anti-inflammatory platform

The collapse of the anti-inflammatory regulatory platform first appears as downregulation of Tregs and ILC2s. Visceral adipose tissue (VAT)-resident Tregs decline markedly in both number and function in obesity (41). One key mechanism is impairment of the cholesterol biosynthesis pathway: Treg-specific deletion of SREBP2 markedly reduces the ST2^hi^ VAT Treg subset and worsens insulin resistance (43). VAT dendritic cells directly suppress Treg differentiation in obesity by downregulating the IL-33 axis (44). As a major source of IL-5/IL-13, ILC2s help maintain M2 macrophage polarization and adipose tissue homeostasis, and both their number and function are markedly impaired in obese VAT (45). Treg subsets co-evolve dynamically with diet and age, indicating that the collapse of the anti-inflammatory platform is a cumulative, time-dependent process (38). CD8^+^ effector memory T cells and Th1 cells expand in obese VAT, and their secreted IFN-γ in turn suppresses the Treg/ILC2/M2 axis (40). From the perspective of the adipose immune atlas, collapse of the anti-inflammatory platform together with expansion of the pro-inflammatory platform constitutes a cellular landscape phase transition in obesity (37).

#### 4.2.2 EPA/DHA → defective SPM synthesis

The second node is interruption of the SPM synthesis pathway. SPMs are generated from the ω-3 polyunsaturated fatty acids EPA and DHA through enzymes such as 15-LOX and 5-LOX, and under physiological conditions they actively drive the resolution of acute inflammation (62). In obesity, SPM levels in adipose tissue and the circulation decline relative to upstream pro-inflammatory mediators, indicating that defective SPM synthesis or release is a core element of resolution failure (63). In obesity–insulin resistance models, SPMs promote M2 macrophage polarization, enhance efferocytosis, suppress the NLRP3 and NF-κB pathways, and activate AMPK and IRS-1/PI3K/Akt signaling (63). Based on these multiple actions, SPMs have themselves become a potential new target for intervention in the obesity–inflammation–metabolic disease comorbidity (62).

#### 4.2.3 Impaired efferocytosis and accumulation of cell debris

The third node is failure to clear apoptotic cells. Under physiological conditions, M2 macrophages clear apoptotic adipocytes through efferocytosis, which both prevents secondary necrosis and amplification of inflammation and initiates a pro-resolving phenotype through the metabolites of engulfment (64). In the obesity–lipotoxic environment, macrophages persistently exposed to excess lipid show impaired efferocytosis, and apoptotic cells and debris accumulate continuously in adipose tissue, forming crown-like structures and releasing DAMPs (64). Impaired efferocytosis and reduced SPM production form a mechanistic positive feedback, because SPMs themselves enhance efferocytosis while the products of efferocytosis serve as substrates for SPM synthesis (63).

#### 4.2.4 SPMs are both anti-inflammatory and analgesic signals

SPMs—particularly resolvins and maresins—also possess potent analgesic properties, a feature that unifies resolution failure and pain sensitization at the molecular level. They act through G-protein-coupled receptors on nociceptive neurons, including GPR18, ALX/FPR2, and GPR37, to lower TRPV1/TRPA1 activity and to suppress glutamate release and spinal pain transmission (15). Loss of SPM receptors alone aggravates the resolution defect and prolongs pain (15). Defective SPM synthesis is therefore a key molecular interface in the obesity–pain comorbidity, and it suggests that supplementation with SPMs or their analogs may simultaneously break the dual failure of resolution and analgesia (15).

### 4.3 Systemic low-grade chronic inflammation and the hepatic acute-phase response

When local inflammation cannot resolve, IL-6 enters the circulation and carries the signal to the liver, initiating a body-wide axis centered on the acute-phase response.

#### 4.3.1 The adipose → liver IL-6 → CRP/SAA acute-phase axis

Adipose-derived IL-6 enters the circulation, acts on the hepatic IL-6R/STAT3 pathway, and drives the production of CRP and SAA (47). In obesity, adipose tissue continuously secretes low levels of TNF-α, IL-1β, and IL-6, so serum hs-CRP often shows a sustained low-grade elevation of 3–10 mg/L (47). SAA is also secreted directly by obese adipocytes; as an inflammatory adipokine, it participates in hepatic HDL remodeling and promotes macrophage activation and monocyte chemotaxis by binding LOX-1 and TLR2/4 (65). During obesity, the liver continuously sustains lipid overload and endoplasmic reticulum stress, and the latter aggravates hepatocyte dysfunction and inflammatory gene transcription through the triple unfolded protein response (UPR) pathways PERK/IRE1α/ATF6 (66).

#### 4.3.2 LGCI-driven increase in cardiovascular risk

Increased cardiovascular risk is the most clinically meaningful downstream outcome of LGCI. A two-sample Mendelian randomization study using IL6/IL6R locus instrumental variables confirmed that IL-6 signaling is causally and positively associated with the risk of coronary artery disease, atrial fibrillation, and T2D (67). A systematic review further indicated that elevated serum IL-6 is associated with the risk of peripheral artery disease, myocardial infarction, and heart failure, and that elevated IL-1β is associated with poor prognosis in coronary heart disease and heart failure (68). SAA and CRP are not merely passive inflammatory markers but active pro-atherogenic molecules; SAA can displace apoA-I and thereby reduce the antioxidant and reverse-cholesterol-transport functions of HDL (65).

#### 4.3.3 LGCI-driven increase in T2D risk

Another key outcome of LGCI is an increased risk of T2D. The NLRP3–IL-1β axis is a core driver of obesity-to-T2D progression: IL-1β translocates NF-κB to the nucleus, drives JNK/IKK activation, and causes IRS-1 serine phosphorylation (33). NLRP3 activation within pancreatic β cells directly impairs their secretory function and accelerates the transition from insulin resistance to T2D (33). Metabolic aging of innate immune cells (inflammaging) further amplifies the pro-inflammatory capacity of monocytes and macrophages as age and obesity advance (51). Neutrophils participate in systemic inflammatory amplification through the secretion of elastase and myeloperoxidase, and their ratio to lymphocytes (NLR) has become a measurable clinical indicator for predicting cardiovascular events and incident T2D (46).

#### 4.3.4 Epigenetic memory and the “weight loss does not end LGCI” phenomenon

LGCI often manifests clinically as a phenomenon in which inflammation is difficult to eliminate through weight loss. A history of obesity can imprint persistent epigenetic changes in murine myeloid cells, so that these cells overrespond to a secondary inflammatory stimulus even after body weight has returned to normal (13). Adipose tissue itself also retains an epigenetic imprint of obesity, and adipocytes that have experienced obesity still display transcriptomic and DNA-methylation scars after weight loss (14). Multi-omics CITE-seq studies further show that obesity-induced imprints in adipose immune cells do not fully resolve after weight loss (54). This mechanism integrates the local positive feedback loop and systemic resolution failure along the temporal dimension, so that LGCI persists at a low level even after body weight normalizes (13).

#### 4.3.5 An integrated perspective and directions for clinical translation

Taken together, the local positive feedback loop and systemic resolution failure are not two independent threads but two faces of a single closed loop. At the local level, the bidirectional paracrine pathway between adipocytes and macrophages, together with NLRP3/NF-κB signaling, locks in chronic inflammation (59). At the systemic level, the IL-6/CRP/SAA axis transmits adipose inflammatory signals to the liver and the whole body (47). Epigenetic memory provides temporal persistence to this loop, allowing LGCI to flare again after weight loss (13). This integrated framework maps directly onto the target-organ injury discussed later, and it provides clear molecular targets for clinical interventions aimed at breaking the loop, including IL-1 antagonism, SPM supplementation, NLRP3 inhibition, and CCR2 blockade (33).

## 5 From inflammation to pain: three-tier sensitization and neuro-immune axis dysfunction

Earlier sections established that obese adipose tissue escalates local inflammation into systemic low-grade chronic inflammation (LGCI), continuously releasing TNF-α, IL-1β and IL-6 into the circulation. This section addresses a key downstream question. How are these circulating and tissue-level pro-inflammatory signals translated into the clinical phenotype of “pain”?

Obesity activates neuro-immune circuits through the saturated-fatty-acid–TLR4 and NLRP3–IL-1β pathways and induces pain directly (35). Excess nutrition and elevated cortisol activate microglia in parallel. Neuroinflammation therefore becomes a shared substrate for metabolic disease, chronic pain and cognitive dysfunction (69). Circulating cytokines also cross a compromised blood–brain barrier and amplify central neuroinflammation (70).

**Figure 4:**
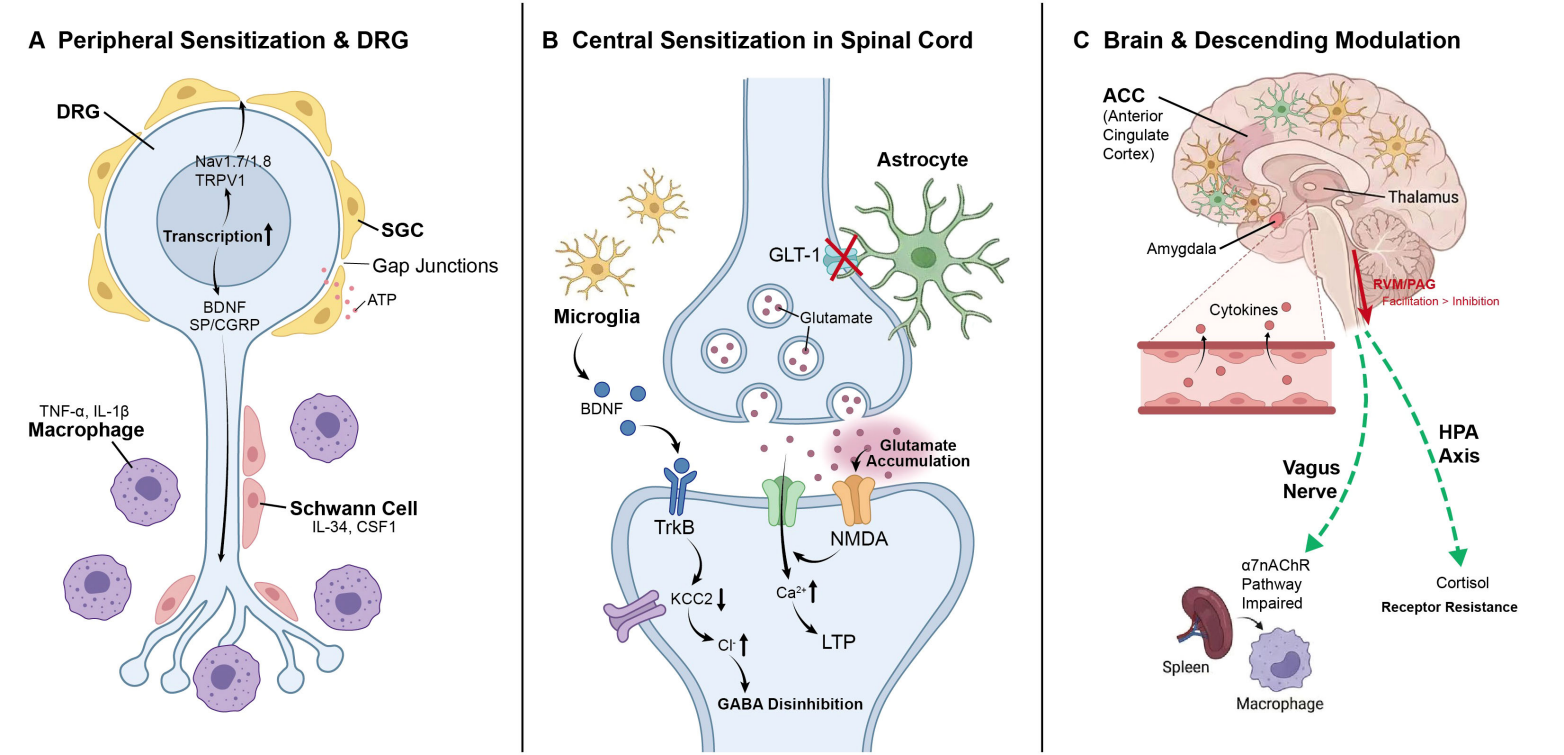
Three-tier pain sensitization mediated by chronic inflammation: from peripheral nociceptor sensitization to central modulation. **(A)** Peripheral Sensitization and the DRG Neuro-Immune Unit. Within the dorsal root ganglion (DRG), macrophages, Schwann cells (releasing interleukin-34, IL-34, and colony-stimulating factor 1, CSF1), and satellite glial cells (SGCs) release TNF-α, IL-1β, and adenosine triphosphate (ATP). Gap junction–mediated coupling amplifies these signals across the neuro-immune unit. In primary sensory neurons, voltage-gated sodium channels Na_v_1.7 and Na_v_1.8 together with transient receptor potential vanilloid 1 (TRPV1) are transcriptionally upregulated, while anterograde and retrograde transport of brain-derived neurotrophic factor (BDNF), substance P (SP), and calcitonin gene-related peptide (CGRP) is enhanced, collectively lowering the nociceptor activation threshold. **(B)** Central Sensitization in the Spinal Cord. In the spinal dorsal horn, activated microglia release BDNF, which acts on second-order neuronal tropomyosin receptor kinase B (TrkB) to downregulate the K^+^–Cl^-^ co-transporter KCC2, causing intracellular chloride (Cl^-^) accumulation and inverting GABAergic inhibition into GABA disinhibition. In parallel, astrocyte-expressed glutamate transporter GLT-1 is downregulated, leading to synaptic glutamate accumulation, excessive N-methyl-D-aspartate (NMDA) receptor activation, and Ca^2+^ influx that induces long-term potentiation (LTP) — the synaptic basis of “pain memory.” **(C)** Brain Remodeling and Descending Modulation. The anterior cingulate cortex (ACC)–amygdala–thalamus circuit is modulated by peripheral inflammatory cytokines that cross the compromised blood–brain barrier. Descending control mediated by the rostral ventromedial medulla (RVM)/periaqueductal gray (PAG) shifts from inhibition toward facilitation. The cholinergic anti-inflammatory pathway, comprising the vagus nerve, α7 nicotinic acetylcholine receptor (α7nAChR), and splenic macrophages, becomes impaired. Sustained hypothalamic–pituitary–adrenal (HPA) axis activation produces glucocorticoid receptor resistance, abolishing cortisol’s anti-inflammatory braking effect. Arrow directionality reflects experimentally supported causal or signaling relationships rather than purely visual flow; bidirectional arrows are used only where reciprocal regulation has been demonstrated. DRG, dorsal root ganglion; IL-34, interleukin-34; CSF1, colony-stimulating factor 1; SGC, satellite glial cell; TNF-α, tumor necrosis factor-alpha; IL-1β, interleukin-1β; ATP, adenosine triphosphate; Na_v_, voltage-gated sodium channel; TRPV1, transient receptor potential vanilloid 1; BDNF, brain-derived neurotrophic factor; SP, substance P; CGRP, calcitonin gene-related peptide; TrkB, tropomyosin receptor kinase B; KCC2, K^+^–Cl^-^ co-transporter 2; GLT-1, glutamate transporter 1; NMDA, N-methyl-D-aspartate; LTP, long-term potentiation; ACC, anterior cingulate cortex; RVM, rostral ventromedial medulla; PAG, periaqueductal gray; α7nAChR, α7 nicotinic acetylcholine receptor; HPA, hypothalamic–pituitary–adrenal.

These observations support a bottom-up three-tier model (**Figure 4**): peripheral sensitization in the dorsal root ganglion (DRG; **Figure 4A**) → spinal central sensitization (**Figure 4B**) → cortical and brainstem remodeling with descending-control imbalance (**Figure 4C**). The apex of the loop is failure of the vagal–HPA neuro-immune axis. Pain then loses its self-terminating mechanism. The framework explains why weight loss alone often fails to abolish pain. Once sensitization is locked into spinal and cortical synapses, reversal of peripheral inflammation is no longer sufficient (71).

### 5.1 Peripheral sensitization and the DRG neuro-immune unit

The DRG is the first relay of nociceptive transmission. Primary sensory neurons here do not act in isolation. They form a tightly coupled “neuro-immune unit” with satellite glial cells (SGCs), Schwann cells and macrophages. This unit translates metabolic-inflammatory signals into elevated neuronal excitability.

#### 5.1.1 The DRG–SGC unit: Kir4.1, gap junctions and ATP/purinergic signaling

SGCs ensheath sensory-neuron somata and represent the peripheral counterpart of central astrocytes. The two cell types share molecular markers and maintain neuronal homeostasis under physiological conditions. After injury or inflammation, SGCs lose homeostatic function and acquire pro-inflammatory features, a state termed “gliopathy” (72).

Three molecular changes drive this transition. First, the inwardly rectifying K^+^ channel Kir4.1 is down-regulated, extracellular K^+^ accumulates, and the neuronal depolarization threshold falls. Second, connexin 43 (Cx43)-mediated SGC–SGC and neuron–SGC gap-junction coupling is enhanced. Third, SGC sensitivity to ATP rises, GFAP is up-regulated, and cytokine release increases. Activated SGCs release ATP, TNF-α, IL-1β and chemokines that amplify firing of adjacent neurons through paracrine action (73). In the spared nerve injury (SNI) model, GFAP^+^ glial cells in the DRG increase in number and cluster around injured neurons. The same population includes non-myelinating Schwann cells, so GFAP is not an absolute SGC marker (74). The ATP–purinergic–cytokine circuit is molecularly homologous to the adipose-tissue NLRP3–IL-1β axis described in Section 4.

#### 5.1.2 The Schwann cell–macrophage cascade: IL-34/CSF1R and TNF-α/IL-1β

A second amplifier of peripheral sensitization is the Schwann cell–macrophage cascade (**Figure 4A**, “Schwann Cell — IL-34, CSF1”). Peripheral nerve injury reprograms Schwann cells, which then secrete IL-34. IL-34 engages colony-stimulating factor 1 receptor (CSF1R) on macrophages. Macrophages infiltrate the DRG, proliferate and activate P38MAPK, NF-κB and the NLRP3 inflammasome. The recruited macrophages release TNF-α and IL-1β, which feed back onto sensory neurons. A “Schwann cell–macrophage–neuron” pro-inflammatory cascade is thereby established (75).

DRG-resident and infiltrating macrophages contribute to both the initiation and the maintenance of neuropathic pain. Their depletion reduces the onset and persistence of hyperalgesia (76). In experimental autoimmune neuritis, DRG macrophage infiltration correlates directly with hyperalgesia. This finding confirms macrophages as a tractable peripheral target (77).

#### 5.1.3 Ion-channel remodeling: Na_V_1.7/1.8, TRPV1 and transcriptional upregulation

The pro-inflammatory milieu ultimately rewrites neuronal electrical properties (**Figure 4A**, “Na_V_1.7/1.8, TRPV1, Transcription↑”). Voltage-gated sodium channels Na_V_1.7 (*SCN9A*) and Na_V_1.8 are selectively enriched in nociceptors. They govern sub-threshold amplification and the rising phase of the action potential, respectively. Both are core targets for inflammatory pain (78).

The clinical relevance of Na_V_1.8 has been validated in large randomized controlled trials. The selective Na_V_1.8 inhibitor suzetrigine (VX-548) significantly outperformed placebo for acute pain (79). Suzetrigine has now been approved for moderate-to-severe acute pain. It is the first non-opioid oral analgesic with a novel mechanism in decades (80). It is worth being precise about what these trials show: these studies evaluated moderate-to-severe acute pain, not the chronic obesity-related phenotypes that are the focus of this review. Reading suzetrigine, or Na_v_1.8 inhibition more broadly, as an analgesic strategy for chronic obesity-related pain therefore goes beyond the existing evidence base and needs dedicated chronic-pain trials before this can be translated into clinical practice. The transient receptor potential vanilloid 1 (TRPV1) channel is up-regulated and sensitized after inflammation and nerve injury. Its sensitization acts as a master switch for inflammatory and neuropathic pain (34). TRPV1 activity is governed by the NLRP3–IL-1β axis. IL-1β directly sensitizes TRPV1, and NLRP3 activation further lowers the pain threshold (12).

#### 5.1.4 Neuropeptides and neurogenic inflammation: CGRP, SP and BDNF

Sensitized nociceptor terminals release calcitonin gene-related peptide (CGRP) and substance P (SP). CGRP drives long-lasting vasodilation. Together with SP it triggers plasma extravasation. CGRP also contributes to peripheral and central sensitization through sensitized primary and secondary afferents (81). On the somatic side, DRG neurons up-regulate brain-derived neurotrophic factor (BDNF). BDNF, CGRP and SP are transported centrally to the spinal dorsal horn (82). Peripheral sensitization is therefore no longer a local event. It becomes the source of an “amplified signal” delivered to the spinal cord and transitions naturally into central sensitization.

#### 5.1.5 Extending the framework to the trigeminal system

The peripheral sensitization machinery laid out above carries over, with cell-type-specific adaptations, to the trigeminal system — which is what most directly concerns obesity-related migraine and orofacial pain. Trigeminal ganglion (TG) neurons run the same nociceptive hardware as DRG neurons (Na_v_ 1.7/1.8, TRPV1, CGRP), but they sit in a distinct neuro-immune environment built around the trigeminovascular afferents innervating the dura and pial vessels.

Three obesity-relevant inputs feed this system. First, systemic LGCI delivers circulating IL-1β, IL-6 and TNF-α to TG neurons through the same NLRP3–IL-1β axis active in the DRG, and TG satellite glial cells undergo the gliopathic reprogramming described in 5.1.1 (12, 73). Second, adipokines such as leptin are elevated in obesity and have been proposed, on the basis of preclinical observations, to sensitize trigeminovascular afferents and modulate cortical excitability — a candidate but as-yet-unvalidated molecular bridge between visceral adiposity and migraine susceptibility that requires further mechanistic study. Third, CGRP release at trigeminovascular terminals drives durovascular neurogenic inflammation, and the clinical efficacy of CGRP-pathway antibodies in migraine — including in patients with metabolic comorbidity — makes this circuit a tractable target inside the MIN framework (81).

Including the trigeminal system here closes the loop between the obesity–migraine link mentioned in Section 1.2 and the peripheral sensitization machinery developed throughout Section 5.1.

### 5.2 Spinal central sensitization

The spinal dorsal horn is the second relay. Sustained peripheral input and local glial activation reshape dorsal-horn synapses so that non-noxious input now evokes pain (allodynia). The mechanisms group into three interleaved axes: collapse of inhibition, impaired glutamate clearance and potentiated excitation.

#### 5.2.1 Microglial P2X4R/P2X7R–BDNF–TrkB–KCC2 and GABA disinhibition

Peripheral nerve injury over-activates spinal microglia. Activated microglia signal through BDNF–TrkB and down-regulate the neuronal K^+^–Cl^-^ co-transporter KCC2. Intracellular Cl^-^ accumulates and the transmembrane anion gradient collapses. GABA- and glycine-mediated inhibitory currents shift from hyperpolarizing to depolarizing — the “GABA disinhibition” annotated at the base of **Figure 4B** (83).

P2X7R is another key purinergic receptor. On microglia, ATP-driven P2X7R activation increases Ca^2+^ influx and unbalances M1/M2 polarization. Cytokine release is amplified, and P2X7R becomes a druggable target (84). The same spinal microglial cascade drives hyperalgesia in metabolic-neuropathy models such as diabetes. This finding links metabolic derangement directly to central sensitization (85).

#### 5.2.2 Astrocytic P2Y1R up-regulation and glutamate accumulation

The “GLT-1 (red cross) → Glutamate Accumulation” element in **Figure 4B** captures resolution failure at the spinal level. Astrocyte-expressed glutamate transporter GLT-1 normally clears synaptic-cleft glutamate. Its expression and function fall in pain states. Glutamate accumulates and postsynaptic excitation rises. In parallel, TNF-α up-regulates astrocytic P2Y1 purinergic receptor (P2Y1R). P2Y1R drives A1/A2 reactive polarization, intensifies Ca^2+^ signaling and promotes Ca^2+^-dependent glutamate release. A positive-feedback “cytokine–purinergic–neuron” loop is established (86). Bidirectional cross-talk between microglia and astrocytes amplifies and sustains this loop. It forms the cellular basis for the transition of central sensitization from “reversible” to “consolidated” (87).

#### 5.2.3 NMDA–Ca^2+^–LTP and “pain memory”

Glutamate accumulation strongly activates NMDA receptors (**Figure 4B**, “NMDA → Ca^2+^↑ → LTP”). NMDA-mediated Ca^2+^ influx engages CaMKII, PKC and ERK. These kinases induce long-term potentiation (LTP) of dorsal-horn excitatory transmission. Epigenetic and synaptic-structural changes “embed” pain into the network. Spinal dorsal-horn LTP is regarded as a form of “pain memory” and constitutes the synaptic basis of the acute-to-chronic transition (88). BDNF–TrkB signaling mediates disinhibition and also enhances NMDA-mediated excitatory transmission. Disinhibition and excitation thereby reinforce each other (83). Such convergent plasticity locks pain into the spinal network and allows it to persist after the original inflammatory stimulus subsides (71).

### 5.3 Supraspinal remodeling and neuro-immune axis failure

The pain pathway extends further within the brain. Ascending signals are relayed by the thalamus to the anterior cingulate cortex (ACC), amygdala and adjacent regions, generating the affective–cognitive dimension of pain. The brain modulates peripheral inflammation and spinal excitability through the RVM/PAG descending system and the vagal–HPA neuro-immune axis. When both control systems fail, pain escalates from a symptom into a self-sustaining disease state.

#### 5.3.1 Thalamus, ACC and amygdala: the affective–cognitive dimension

The thalamus relays ascending nociceptive signals to the cortex. Chronic pain remodels thalamic function. HCN2 channels in the ventral posterolateral nucleus are up-regulated, thalamocortical excitability rises, and neuropathic pain is driven (89). The ACC is the cortical hub of the affective–motivational component of pain. ACC synapses undergo LTP in chronic pain. NMDA-receptor activation triggers a Ca^2+^ cascade. AMPA receptor function expands, including unsilencing of silent synapses and GluA1 recruitment. Postsynaptic potentiation is sustained, the affective burden of pain persists, and comorbidity with anxiety and depression is established (90). The parabrachial-to-central-amygdala (PBN→CeA) projection is another critical node. After injury, transmission in this pathway is enhanced and directly mediates injury-induced pain sensitization and the affective–motivational component of pain (91).

#### 5.3.2 RVM/PAG descending-control imbalance: facilitation > inhibition

The red “RVM/PAG (Facilitation > Inhibition)” element in **Figure 4C** marks the pathological tilt in descending control. The periaqueductal-gray (PAG)–rostral-ventromedial-medulla (RVM) pathway is the core of descending pain modulation. It exerts bidirectional facilitatory and inhibitory drive onto the dorsal horn. Under chronic pain and chronic stress, the balance of ON-cell and OFF-cell activity in the RVM is disrupted. Descending facilitation is relatively enhanced and inhibition is reduced. Spinal nociceptive input is therefore amplified rather than damped. The RVM is tightly connected with the ACC, amygdala and other affective–stress regions. This explains why stress and negative affect can objectively worsen pain (92).

#### 5.3.3 Impaired cholinergic anti-inflammatory pathway: vagus–α7nAChR–spleen

The “Vagus Nerve → α7nAChR Pathway Impaired → Spleen Macrophage” arrow in the lower-right of **Figure 4C** depicts failure of the inflammatory reflex. Under physiological conditions, efferent vagal signals reach the spleen via the splenic nerve and release noradrenaline. Noradrenaline stimulates splenic cholinergic T cells to release acetylcholine. Acetylcholine activates α7 nicotinic acetylcholine receptors (α7nAChR) on macrophages and suppresses release of TNF-α and other cytokines. This is the cholinergic anti-inflammatory pathway (CAP). In chronic inflammation and obesity, vagal tone falls, the negative feedback weakens, and systemic inflammation loses its brake (93).

Vagus nerve stimulation (VNS) has been explored on this rationale for chronic pain and inflammatory disorders. Meta-analytic evidence supports a moderate analgesic effect of transcutaneous VNS in chronic pain (94). The anti-inflammatory effect of VNS in humans, however, is not consistent across studies. Efficacy depends on stimulation parameters and indication. Rigorous randomized controlled trials are still needed (95). It is worth being explicit about what each study supports: Costa’s study (94) documents a moderate analgesic effect of transcutaneous VNS in chronic pain, not an anti-inflammatory effect; and Schiweck (95) reports that systemic anti-inflammatory readouts (TNF-α, IL-6, CRP) are inconsistent in human VNS studies. Neither, on its own, justifies a generalized anti-inflammatory claim for VNS in obesity-related chronic pain, and the two roles — analgesia versus systemic anti-inflammation — should be kept separate when VNS is read as a neuro-immune intervention.

#### 5.3.4 HPA-axis dysregulation and glucocorticoid resistance

The “HPA Axis → Cortisol → Receptor Resistance” element on the right of **Figure 4C** marks the second failure of neuro-immune braking. The hypothalamic–pituitary–adrenal (HPA) axis exerts physiological anti-inflammatory action through cortisol. Under sustained chronic inflammation and chronic stress, the glucocorticoid receptor (GR) becomes “resistant”. Cortisol’s anti-inflammatory signaling is impaired, negative feedback fails, and pro-inflammatory cytokines continue to rise (96).

In obesity, prolonged cortisol exposure and altered GR sensitivity act simultaneously on adipose biology and the central nervous system. The stress–obesity–inflammation triad is mutually reinforced (97). This process is tightly coupled with the persistence of LGCI described in Section 4 and amplifies chronic pain through neuroinflammation (69).

#### 5.3.5 Integrated view and translational directions

**Figure 4** integrates the pathogenesis of pain into a continuous chain. The chain runs from periphery to spinal cord to brain and is fed back by the neuro-immune axis. Peripherally, the DRG neuro-immune unit translates metabolic inflammation into neuronal hyperexcitability (75). Spinally, microglia–astrocyte-mediated disinhibition and NMDA–LTP consolidate sensitization into a “pain memory” (88). Centrally, thalamus–ACC–amygdala remodeling, RVM-dependent descending facilitation and vagal/HPA brake failure render pain self-sustaining (92).

This framework maps directly onto multi-level intervention targets that may break the loop. Peripheral targets include IL-34/CSF1R (75), Na_V_1.8 (79) and TRPV1 (34). Spinal targets include the P2X4R/P2X7R–BDNF–TrkB–KCC2 axis (83) and the glutamate-clearance system (86). Central and neuro-immune targets include VNS (94), α7nAChR agonists (93), and supplementation with specialized pro-resolving mediators (SPMs) — molecules that combine anti-inflammatory and analgesic actions (15). In the obesity–pain comorbidity context, combining metabolic intervention with these neuro-immune targets may simultaneously interrupt both the LGCI loop and the three-tier sensitization loop (35).

## 6 Clinical pain phenotypes: a precision classification framework based on metabolic–immune mechanisms

**Figure 5:**
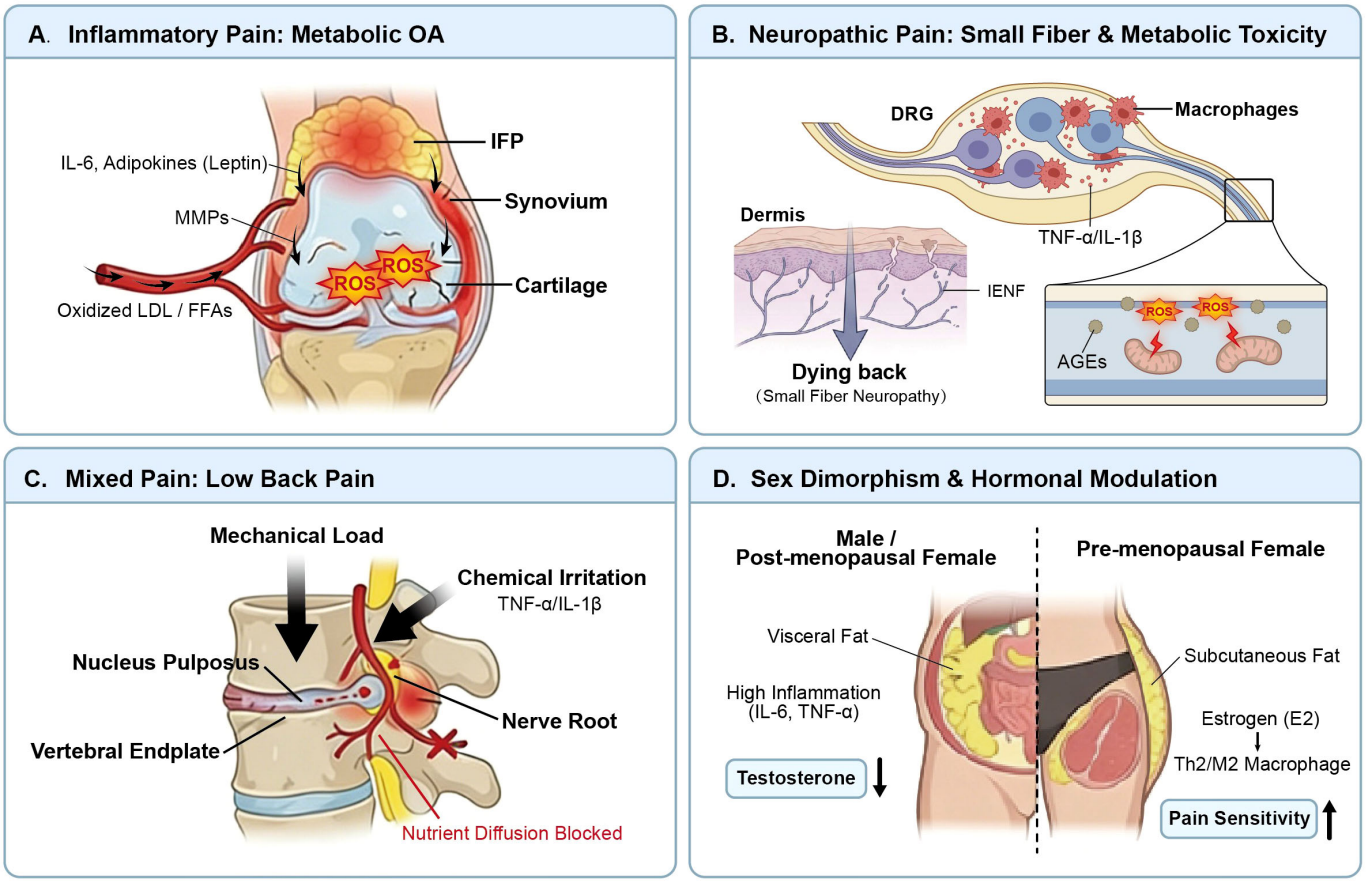
Clinical pain phenotypes and sex dimorphism: a precision classification framework based on metabolic–immune mechanisms. **(A)** Inflammatory Pain — Metabolic Osteoarthritis (OA). Obesity drives the infrapatellar fat pad **(IFP)** to secrete IL-6 and adipokines such as leptin. Oxidized LDL (ox-LDL) and FFAs infiltrate the joint cavity through the vasculature, activating matrix metalloproteinases (MMPs) and inducing ROS accumulation. The combined action of synovitis and lipotoxicity drives cartilage destruction and pain chronification. **(B)** Neuropathic Pain — Small Fiber Neuropathy and Metabolic Toxicity. Advanced glycation end products (AGEs) accumulate in the DRG, causing mitochondrial dysfunction and ROS production. Infiltrating macrophages release TNF-α and IL-1β to sensitize sensory neurons. Intraepidermal nerve fibers (IENFs) undergo characteristic dying-back degeneration, the histological hallmark of small fiber neuropathy. **(C)** Mixed Pain — Chronic Low Back Pain (LBP). Mechanical loading compresses the nucleus pulposus (NP) and vertebral endplate, blocking nutrient diffusion. The degenerated NP secretes TNF-α and IL-1β to chemically irritate the nerve root. Mechanical loading and chemical irritation jointly drive the nociceptive and neuropathic components of LBP. **(D)** Sex Dimorphism and Hormonal Modulation. Males and post-menopausal females exhibit a pro-inflammatory state characterized by visceral adiposity, declining testosterone, and elevated IL-6 and TNF-α. Pre-menopausal females show predominantly subcutaneous adiposity, with estrogen (E2) promoting T helper 2 (Th2) and M2 macrophage polarization toward an anti-inflammatory state, yet paradoxically exhibit higher pain sensitivity — reflecting central neuro-immune mechanisms distinct from peripheral inflammatory status. Arrow directionality reflects experimentally supported causal or signaling relationships rather than purely visual flow; bidirectional arrows are used only where reciprocal regulation has been demonstrated. OA, osteoarthritis; IFP, infrapatellar fat pad; IL-6, interleukin-6; ox-LDL, oxidized low-density lipoprotein; FFAs, free fatty acids; MMPs, matrix metalloproteinases; ROS, reactive oxygen species; AGEs, advanced glycation end products; DRG, dorsal root ganglion; TNF-α, tumor necrosis factor-alpha; IL-1β, interleukin-1β; IENF, intraepidermal nerve fiber; LBP, low back pain; NP, nucleus pulposus; E2, estrogen (17β-estradiol); Th2, T helper 2 cell; M2, M2 macrophage.

Chronic pain is clinically heterogeneous, and traditional anatomical or temporal classifications no longer meet the demands of precision medicine. The interaction between metabolic dysregulation and immune imbalance is a core driver of pain phenotype formation (98). On this basis, chronic pain can be categorized into four representative phenotypes (**Figure 5**): inflammatory pain mediated by metabolic osteoarthritis (OA), neuropathic pain mediated by diabetic small fiber neuropathy (SFN), mixed pain in chronic low back pain (LBP), and sex-dimorphic pain regulated by sex hormones. This section systematically describes the molecular pathology and translational implications of these four phenotypes.

### 6.1 Inflammatory pain phenotype: metabolic osteoarthritis

Metabolic OA is a distinct subtype driven by obesity and metabolic syndrome. The Global Burden of Disease Study 2021 reported that OA affects approximately 595 million individuals worldwide, with cases projected to increase by 60%–100% by 2050. High BMI is a major modifiable risk factor, with an attributable fraction of 20.4% (99). Metabolic dysregulation drives OA through glucolipid disturbance, chronic low-grade inflammation, and oxidative stress, extending well beyond the traditional mechanical loading paradigm (98).

The infrapatellar fat pad (IFP) is the key effector organ in metabolic OA (**Figure 5A**). During OA progression, the IFP develops adipocyte hypertrophy, fibrosis, neovascularization, and macrophage infiltration, secreting pro-inflammatory cytokines and adipokines that shape the joint immune microenvironment and pain signaling (100). Inflammation within the IFP exhibits a clear spatial gradient: the synovial-side inflammatory layer expresses substantially higher levels of IL-1β, TNF-α, IL-8, leptin, and adiponectin than the deeper region, accompanied by predominant B-cell infiltration (101). Leptin, the principal adipokine secreted by the IFP, is elevated in the serum of OA patients and regulates chondrocyte metabolism and macrophage polarization through the JAK/STAT3 pathway (102).

Dyslipidemia-driven lipotoxicity represents another key mechanism. Free fatty acids (FFAs) and oxidized LDL (ox-LDL) stimulate chondrocytes to release IL-6 and TNF-α via the TLR4/NF-κB pathway, upregulate MMP-13 expression, and accelerate type II collagen degradation, thereby establishing a self-sustaining “inflammation–ROS–matrix degradation” microenvironment (103). Synovial IL-1β and TNF-α further upregulate Na_v_1.7 sodium channel expression in sensory neurons, lowering the excitation threshold of nociceptors — a critical step in pain chronification in metabolic OA (103).

### 6.2 Neuropathic pain phenotype: diabetic small fiber neuropathy and metabolic toxicity

Diabetic peripheral neuropathy (DPN) affects 30%–50% of patients with type 2 diabetes mellitus (T2DM). Among patients with DPN, the pooled prevalence of painful DPN (PDPN) reaches 46.7% (95% CI: 41.8%–51.7%) (104), while a separate meta-analysis reports a pooled PDPN prevalence of 33.9% in the diabetic population overall (105). The core pathological feature of PDPN is the selective degeneration of myelinated Aδ and unmyelinated C fibers (**Figure 5B**).

Chronic hyperglycemia drives small fiber degeneration through multiple pathways. Glucolipid dysregulation activates the polyol pathway and promotes the accumulation of advanced glycation end products (AGEs); AGE–RAGE binding then induces oxidative stress and mitochondrial dysfunction in dorsal root ganglion (DRG) neurons (106). Axonal dying-back degeneration is the typical histological change in DPN. Human tissue studies have identified Nageotte nodules in PDPN DRGs — degenerative structures composed of satellite glia and non-myelinating Schwann cells, invaded by Peripherin/Na_v_1.7-positive dystrophic axons — that account for approximately 25% of all neurons (107).

Skin punch biopsy is a key tool for evaluating small fiber degeneration and neurovascular coupling. PDPN patients show a significant decrease in PGP9.5-positive intraepidermal nerve fiber density (IENFD) in thigh skin, alongside abnormally elevated vWF-positive vascular density, indicating neurovascular imbalance (108). Earlier studies in calf skin similarly confirmed that PDPN groups exhibit more pronounced IENFD reduction and vWF elevation than painless DPN groups (109).

Chronic inflammation and immune imbalance are central to PDPN chronification. Local DRG macrophages, activated by FFAs and AGEs, polarize toward the M1 phenotype; the resulting TNF-α, IL-1β, and IL-6 upregulate Na_v_1.3/1.7 sodium channels and TRPV1 function through the p38 MAPK pathway, thereby sensitizing neurons (110). Risk factor analyses grounded in metabolic–immune mechanisms identify age, female sex, elevated BMI, diabetes duration, and nephropathy as independent risk factors for PDPN (104).

### 6.3 Mixed pain phenotype: chronic low back pain

LBP is a mixed pain phenotype combining nociceptive and neuropathic components (**Figure 5C**). Approximately 40% of LBP cases are attributable to intervertebral disc degeneration (IVDD), the core mechanism of which involves the interplay of mechanical loading, chemical inflammation, and impaired nutrient diffusion (111).

Mechanical stress on nucleus pulposus (NP) cells initiates IVDD. Obesity aggravates disc catabolism by increasing intradiscal pressure and by systemically elevating leptin and resistin. A prospective study based on the Northern Finland Birth Cohort 1966 confirmed that the co-occurrence of obesity and lumbar disc degeneration substantially amplifies LBP-related disability (112).

Chemical irritation of the nerve root is the core mechanism of IVDD-related pain. TNF-α and IL-1β secreted by the degenerated NP sensitize nerve roots and promote nerve ingrowth into the normally aneural inner NP, forming a painful neuro-immune interaction (111). The cartilage endplate (CEP) is the main nutrient diffusion route for the NP. Inflammation, mechanical stress, and ferroptosis trigger CEP calcification and microvascular degeneration, blocking nutrient supply (113). This process accelerates NP cell senescence and extracellular matrix breakdown, establishing a “metabolic starvation–inflammation amplification” cycle (114). Accordingly, precision intervention for LBP should target different pathological stages of IVDD: early stages focus on correcting mechanical loading and on anti-inflammation, whereas progressive stages should target CEP reconstruction and neuro-immune modulation (111).

### 6.4 Sex dimorphism and hormonal modulation

Chronic pain prevalence and nociceptive sensitivity are markedly higher in females than in males. This dimorphism involves sex hormone regulation at both peripheral adipose distribution and central neuro-immune pathways (**Figure 5D**) (115).

At the peripheral level, estrogen regulates adipose distribution and systemic inflammation. A meta-analysis revealed that post-menopausal women have significantly higher plasma leptin and adiponectin levels than pre-menopausal women, alongside increased waist circumference, waist-to-hip ratio, and BMI, indicating that the menopausal transition is a critical window for metabolic–inflammatory imbalance (116). In males, age-related testosterone decline is accompanied by visceral fat accumulation and systemic IL-6/TNF-α elevation, forming the metabolic–immune basis of “male menopause” (115).

At the central neuro-immune level, M2 macrophages exert anti-inflammatory and neuroprotective effects and represent key effectors in pain relief, with sex-specific differences in their polarization (117). Preclinical studies reveal markedly distinct sex-specific immune pathways: in male rodents, spinal microglia mediate neuropathic pain maintenance through BDNF signaling, whereas in females, T-lymphocyte-mediated adaptive immunity is the key driver of pain persistence (118). Transcriptomic studies further confirm that male spinal microglia display extensive pro-inflammatory transcriptional changes after nerve injury, while female microglia exhibit a fundamentally different transcriptional pattern (119). This mechanistic divergence carries critical translational implications: microglia-targeted analgesic strategies may show reduced efficacy in female patients (118).

Migraine is another hormone-related pain phenotype. In females, prolactin sensitizes nociceptors and enhances calcitonin gene-related peptide (CGRP) release, mediating female-selective migraine and providing a new target for sex-specific analgesic development (120). To help readers separate clinically mature targets from mechanistically plausible but unproven ones, **Table 1** maps these four phenotypes onto seven dimensions of evidence and intervention. The table is intended as a triage tool: it makes explicit, for each phenotype, what is supported by direct clinical evidence, what is supported only by mechanism or preclinical work, and where the current limitations lie.

**Table 1:** Phenotype-to-mechanism mapping of the four representative obesity-related chronic pain phenotypes, with the supporting EndNote reference numbers listed in the final column.

Phenotype	Metabolic drivers	Immune mediators	Neural mechanisms	Evidence strength	Biomarkers	Candidate interventions	Current limitations	References
Metabolic OA	IFP hypertrophy; FFAs; ox-LDL; leptin/adiponectin imbalance	IL-1β, IL-6, TNF-α; M1 ATMs; B-cell infiltration in IFP	Synovial IL-1β/TNF-α → Na_v_ 1.7 upregulation on sensory neurons	Phase-III RCT (semaglutide, STEP 9, knee OA only) + multiple observational cohorts	Serum leptin, IL-6, MMPs; IFP volume on MRI	GLP-1 RAs; NSAIDs; weight loss; IL-1 antagonism (experimental)	RCT evidence beyond knee OA limited; analgesic effect not cleanly separable from weight effect	8, 33, 98–103
Diabetic SFN/PDPN	Chronic hyperglycemia; AGEs; mitochondrial dysfunction; polyol pathway	DRG M1 macrophages; TNF-α, IL-1β, IL-6; p38 MAPK	Na_v_ 1.3/1.7 ↑; TRPV1 sensitization; Aδ/C-fiber dying-back; Nageotte nodules	Strong human histology (IENFD, vWF) + mechanistic preclinical	IENFD on punch biopsy; QST; skin vWF	Glycemic control; α-lipoic acid; Na_v_ 1.8 inhibitors (acute-pain evidence only); SPM analogs (preclinical)	Glycemic control alone often insufficient; no long-term human RCTs for disease-modifying agents	15, 62, 79, 80, 104–110, 121, 122
Mixed-mechanism LBP	Mechanical load × obesity-driven leptin/resistin; impaired CEP nutrition; ferroptosis	NP-derived TNF-α, IL-1β; nerve ingrowth; macrophage infiltration	Nociceptive (mechanical) + neuropathic (nerve-root irritation, ingrowth) components	Imaging + observational cohorts; mechanistic preclinical	Pfirrmann grade; Modic changes; serum CRP/IL-6	Early: load correction + anti-inflammation; late: CEP repair/neuro-immune modulation (experimental)	Phenotyping not standardized; few mechanism-targeted RCTs	111–114
Sex-dimorphic pain	Sex-hormone-dependent fat distribution; estrogen withdrawal; testosterone decline	Sex-specific microglia vs T-cell pathways; sex differences in cytokine profile	Male: spinal microglia–BDNF–TrkB; Female: T-cell-driven; prolactin–CGRP in migraine	Strong preclinical (rodent); emerging human evidence	Sex-stratified inflammatory panels (CRP, IL-6, leptin)	Sex-specific analgesic strategy; prolactin/CGRP-pathway agents in female migraine	Sex poorly integrated into trial design; microglia/T-cell dichotomy needs human translation	5, 6, 115–120

ATM, adipose tissue macrophage; CEP, cartilage endplate; CRP, C-reactive protein; DRG, dorsal root ganglion; FFA, free fatty acid; GLP-1RA, glucagon-like peptide-1 receptor agonist; IENFD, intraepidermal nerve fiber density; IFP, infrapatellar fat pad; LBP, low back pain; NP, nucleus pulposus; OA, osteoarthritis; PDPN, painful diabetic peripheral neuropathy; QST, quantitative sensory testing; RCT, randomized controlled trial; SFN, small fiber neuropathy; SPM, specialized pro-resolving mediator; vWF, von Willebrand factor.

### 6.5 Translational implications: building a multi-dimensional precision classification framework

Integrating the metabolic–immune evidence across these four clinical phenotypes, precision diagnosis and treatment of chronic pain should rely on multi-dimensional biomarker combinations. The inflammatory OA phenotype can be molecularly stratified using serum leptin, IL-6, and MMPs (103). The PDPN phenotype can be evaluated by IENFD, skin vWF, and serum inflammatory markers (108). The mixed pain features of LBP should be assessed by integrating imaging findings (Pfirrmann grading, Modic changes) with inflammatory biomarkers (111). Sex dimorphism mechanisms should be incorporated into clinical decisions across all pain phenotypes (115). This framework shifts the traditional symptomatic analgesia paradigm toward disease-modifying strategies that target underlying metabolic–immune mechanisms, providing a theoretical basis for developing sex-specific and phenotype-oriented precision analgesia.

## 7 Therapeutic implications: multi-target intervention strategies along the “metabolic–immune–neural” axis

Preceding sections delineated a closed causal loop extending from adipose tissue inflammation to neural sensitization (36). Within this framework, intervention paradigms for obesity–pain comorbidity are evolving from a simple “analgesia plus weight loss” model toward a three-dimensional strategy of metabolic correction, inflammation resolution, and neuroimmune rebalancing (10). Seven representative intervention modalities are organized below by mechanistic hierarchy.

### 7.1 Targeted intervention of the NLRP3/IL-1β pathway

The NLRP3–IL-1β axis is constitutively activated under obesity and insulin resistance and constitutes a core mechanism of adipose tissue inflammation and pancreatic β-cell dysfunction (11). This axis also drives IL-1β and IL-18 release from neurons in the spinal dorsal horn and dorsal root ganglia, directly contributing to neural sensitization (12). Clinically, IL-1 pathway blockade—including the oral NLRP3 inhibitor dapansutrile and IL-1 monoclonal antibodies—significantly improves glycemic control and reduces HbA1c in patients with type 2 diabetes and obesity (33). Preclinically, the selective NLRP3 inhibitor MCC950 attenuates cardiac remodeling and metabolic dysfunction in obese mice and promotes M2 macrophage polarization (123). At the ion-channel level, the first-in-class non-opioid oral analgesic suzetrigine (VX-548), targeting Na_v_1.8, has demonstrated efficacy in phase II/III trials and received FDA approval in early 2025 for moderate-to-severe acute pain (121). Two scope reminders apply here. The clinical IL-1 blockade evidence cited above (33) comes from trials with glycemic and metabolic-syndrome endpoints, not dedicated pain trials, so reading this axis as an analgesic target in obesity-related chronic pain is for now a mechanism-based inference. And the suzetrigine approval (121) is specifically for moderate-to-severe acute pain — extending it to the chronic obesity-related phenotypes that this review is about would go beyond the trial evidence, and the chronic-pain trials of Na_v_ 1.8 inhibition in metabolic-pain phenotypes have not yet been done.

### 7.2 Specialized pro-resolving mediator analogs

SPMs—including resolvins, protectins, maresins, and lipoxins—are generated from ω-3 PUFAs through 15-LOX/5-LOX enzymatic activity. They act on immune cells and sensory neurons via G protein-coupled receptors such as GPR18, ALX/FPR2, GPR32, and GPR37, producing dual anti-inflammatory and analgesic effects. Loss of SPM receptors aggravates impaired resolution and prolongs pain duration (15). Circulating SPM levels relative to pro-inflammatory mediators are markedly reduced in obese individuals, representing a key molecular event in resolution failure. RvD1, RvE1, and Maresin 1 produce robust analgesia in multiple neuropathic and postoperative pain models and have emerged as promising candidates for metabolic–pain comorbidity (62).

### 7.3 GLP-1 receptor agonists

GLP-1RAs represent the intervention strategy with the strongest clinical evidence along the metabolic-correction-to-analgesia axis. In 407 patients with obesity and moderate knee osteoarthritis, the STEP 9 trial showed that once-weekly semaglutide 2.4 mg over 68 weeks significantly reduced both body weight and WOMAC pain scores and improved physical function (8). This is the strongest piece of direct clinical evidence in the review, but it is also the most narrowly scoped: STEP 9 (8) enrolled patients with concurrent obesity and knee OA only. Generalizing the WOMAC-pain effect to other obesity-related phenotypes — diabetic neuropathy, mixed-mechanism low back pain, fibromyalgia, migraine — goes beyond what reference 8 supports, and phenotype-specific trials are needed before semaglutide can be positioned as a general analgesic in the obesity–pain comorbidity. In 17, 604 patients with obesity and cardiovascular disease without diabetes, the SELECT trial demonstrated a 20% reduction in major adverse cardiovascular events with semaglutide (124). In SURMOUNT-1, the GIP/GLP-1 dual agonist tirzepatide produced 20.9% weight loss at 72 weeks (125). Beyond weight reduction, GLP-1RAs directly suppress microglial activation in rodent neuropathic pain models, reducing TNF-α, IL-6, and IL-1β expression (126). A systematic review of 42 studies—covering osteoarthritis and inflammatory pain, headache, neuropathic pain and diabetic neuropathy, and visceral pain with irritable bowel syndrome—indicates that the analgesic effect of GLP-1RAs is consistent across these disease categories (127).

### 7.4 Omega-3 polyunsaturated fatty acids

ω-3 PUFAs serve as substrates for SPM biosynthesis and reduce PGE2 and LTB4 generation through competitive inhibition of arachidonic acid metabolism (15). A meta-analysis of 41 randomized controlled trials (n=3, 759) reported moderate analgesic effects of ω-3 supplementation in chronic pain, with the effect strengthening to a standardized mean difference of –0.83 at 6 months (128). In rheumatoid arthritis, a meta-analysis of 18 randomized controlled trials (n=1, 018) confirmed that ω-3 supplementation significantly reduces triglycerides, tender joint count, and the ω-6/ω-3 ratio (129).

### 7.5 Exercise and metabolic weight loss

Exercise exerts systemic anti-inflammatory effects through myokines released by contracting skeletal muscle: acute exercise induces muscle-derived IL-6, which subsequently elicits IL-1Ra and IL-10 production, and moderate-intensity aerobic exercise lowers hs-CRP in obese individuals (61). Bariatric surgery provides stronger anti-inflammatory evidence. A 4-year cohort study of 163 patients reported sustained reductions in CRP, hs-CRP, leukocyte counts, and ferritin following laparoscopic sleeve gastrectomy and Roux-en-Y gastric bypass, with the magnitude of improvement linearly related to weight loss (130).

### 7.6 Vagus nerve stimulation

The vagus–α7nAChR–splenic circuit constitutes a classical inflammatory reflex, with vagal tone diminished in obesity and chronic stress (9). VNS exerts dual effects through the cholinergic anti-inflammatory pathway and the descending noradrenergic system (131). A systematic review and meta-analysis provides moderate-quality evidence for analgesic effects of transcutaneous vagus nerve stimulation (tVNS) in chronic pain (94). In contrast, another systematic review and meta-analysis of human studies found no consistent reduction in systemic inflammatory markers (TNF-α, IL-6, CRP) with VNS, indicating that its anti-inflammatory effects depend strongly on stimulation parameters and indication, and require validation through rigorous randomized controlled trials (95). To put this in plain terms: Costa’s study (94) supports an analgesic, not an anti-inflammatory, effect; Schiweck (95) documents the inconsistency of the anti-inflammatory readout in humans. Neither, on its own, justifies framing VNS as a generalized neuro-immune intervention for obesity-related chronic pain, and the analgesic and anti-inflammatory claims should be reported separately rather than collapsed together.

### 7.7 Epigenetic interventions

After weight loss, myeloid cells and adipose tissue retain persistent pro-inflammatory epigenetic imprints, providing the molecular basis for the “weight loss without pain relief” phenomenon. In mouse models, prior obesity inscribes durable epigenetic changes in innate immune cells, producing exaggerated responses to secondary inflammatory challenge even after weight recovery (13). Adipocytes themselves retain transcriptomic and epigenetic scars of obesity and respond more strongly to repeated high-fat diet challenge; this phenomenon has been confirmed by single-nucleus RNA sequencing in both human and mouse adipose tissue (14). HDAC3 inhibition, SIRT1 activation, and H3K4me3 modulation provide conceptual targets for epigenetic reversal in metabolic disease, although interventions at this level remain largely preclinical (132).

### 7.8 An integrated multi-pathway perspective

The seven strategies above are not independent options but should be combined according to the patient’s metabolic–immune phenotype (47). Breaking the closed loop requires synchronous intervention at three levels—peripheral (NLRP3/IL-1β, Na_v_1.8), spinal (microglia-mediated disinhibition), and central (VNS, α7nAChR)—while GLP-1RAs and bariatric surgery interrupt the temporal recurrence cycle (35).

## 8 Conclusions and perspectives

### 8.1 Inflammation–pain as a common terminal pathway of metabolic disease

Low-grade chronic inflammation driven by obesity and metabolic syndrome forms the shared substrate for multiple complications—type 2 diabetes, cardiovascular disease, and osteoarthritis—and constitutes a core mechanism of chronic pain (3). A scoping review of 28 observational studies confirmed that metabolic syndrome is significantly enriched in populations with migraine, spinal pain, fibromyalgia, and general chronic pain, with a bidirectional relationship between the two (7). Epidemiological evidence supports this view: the global obese population exceeded one billion in 2022 (1); GBD 2021 projections estimate that approximately 3.8 billion adults will be overweight or obese by 2050 (2); and GBD 2021 analyses of osteoarthritis project further increases in case counts by 2050, with elevated BMI as the leading modifiable attributable risk factor (99).

A continuous causal chain links adipocyte stress, immunometabolic reprogramming, positive feedback loops with resolution failure, three-level sensitization, and clinical phenotypic divergence. Within this chain, sensitization and desensitization of peripheral nociceptors—particularly TRPV1—constitute a critical pain switch (34). For the subset of chronic pain phenotypes driven or amplified by metabolic dysregulation, reading the condition as a neuroimmune consequence of unresolved metabolic inflammation has clear translational implications. We are not arguing that this framework covers chronic pain in general — pain is mechanistically heterogeneous, and conditions such as primary neuropathic syndromes, structural musculoskeletal pain without metabolic comorbidity, and centralized pain states share only partial overlap with the MIN framework. The framework is most useful where there is epidemiological, biochemical, or imaging evidence of a metabolic–inflammatory contribution, and it should inform phenotype-specific — not universal — decisions about diagnosis and treatment. A meta-analysis confirmed that individuals with obesity or excess weight report significantly greater pain intensity than normal-weight individuals (4).

### 8.2 Individualized strategies based on multi-omics and epigenetic stratification

Precision intervention requires a clinical decision framework combining multidimensional biomarkers with epigenetic stratification. Key metabolic indicators include serum leptin, IL-6, CRP/SAA, and the ceramide lipidome; ceramides have been established as independent risk markers for cardiovascular events, T2DM, and metabolic syndrome (25). The immune dimension should incorporate single-cell phenotyping of the visceral adipose Treg/M2 axis (41). The neural dimension should integrate intraepidermal nerve fiber density (IENFD) with quantitative sensory testing (QST); IENFD has been established as a key reference indicator for the diagnosis of small fiber neuropathy by systematic review and meta-analysis (122).

Sexual dimorphism should function as a critical variable across all clinical decisions. A study of 32, 409 abdominal MRIs from the UK Biobank showed that the association between visceral and subcutaneous adipose tissue and multisite chronic pain has a larger effect size in women (5). A cross-sectional analysis from the All of Us cohort further confirmed significant sex differences in the combined predictive capacity of waist-to-hip ratio with CRP, IL-6, and leptin for chronic pain (6).

### 8.3 Limitations and future directions

Four key gaps remain in this framework. First, multi-omics data from human tissues—particularly DRG, spinal cord, and adipose tissue—are scarce, limiting translation of mechanistic evidence from rodents to humans. Second, the long-term safety and optimal dosing of SPM analogs, NLRP3 inhibitors, and VNS in humans remain insufficiently characterized (13). Third, whether epigenetic memory can be fully reversed by existing interventions, and the temporal window for such reversal, remain unclear (132). Fourth, the molecular mechanisms of sexual dimorphism are not yet adequately integrated into clinical trial design (6).

Three research priorities follow from these gaps: building large-scale multi-omics databases from human tissues to characterize obesity–pain comorbidity; conducting head-to-head randomized controlled trials to evaluate the relative efficacy of distinct mechanistic strategies in defined phenotypic subgroups; and developing epigenetic agents capable of reversing trained immunity. As chronic pain is increasingly recognized as the neuroimmune terminal complication of metabolic disease, its management is entering a new precision era defined by multi-target, cross-level, sex-specific, and epigenetically reversible approaches.

A final calibration note that applies across the review. Several of the chains of reasoning developed above lean on evidence from outside the chronic-pain literature, and it is worth being explicit about which inference rests on what. The work of Hata et al., Hinte et al., Caslin et al. and Cottam et al. directly supports persistent epigenetic and inflammatory memory after weight loss in myeloid cells and adipose tissue, but the link to persistent clinical pain after metabolic correction is a mechanism-based extrapolation that still needs human pain-endpoint trials (13, 14, 53, 54). Meier et al. support NLRP3/IL-1 blockade for metabolic and glycemic endpoints; reading the axis as an analgesic target in obesity-related pain is currently inferred from mechanism rather than from dedicated pain RCTs (33). The trials reported by Jones et al., Peshin et al. and Hang Kong et al. establish Na_v_ 1.8 inhibition for acute pain — not for the chronic phenotypes that are this review’s subject (79, 80, 121). Costa et al. and Schiweck et al. support, respectively, a moderate analgesic effect of VNS and the inconsistency of its systemic anti-inflammatory effect in humans; together they do not justify a generalized neuro-immune claim (94, 95). The strongest piece of direct clinical evidence in this review is the STEP 9 trial reported by Bliddal et al., and that evidence is specifically about knee OA pain — it should not be extended to other obesity-related pain phenotypes without dedicated trials (8). Future work should target each of these specific extrapolations with phenotype-defined, pain-endpoint randomized trials before any of them is brought into clinical guidance.
